# Conformational change of RNA-helicase DHX30 by ALS/FTD-linked FUS induces mitochondrial dysfunction and cytosolic aggregates

**DOI:** 10.1038/s41598-022-20405-2

**Published:** 2022-09-26

**Authors:** Ryota Hikiami, Toshifumi Morimura, Takashi Ayaki, Tomoyuki Tsukiyama, Naoko Morimura, Makiko Kusui, Hideki Wada, Sumio Minamiyama, Akemi Shodai, Megumi Asada-Utsugi, Shin-ichi Muramatsu, Takatoshi Ueki, Ryosuke Takahashi, Makoto Urushitani

**Affiliations:** 1 Department of Neurology, Shiga University of Medical Science, Seta-Tsukinowa-Cho, Otsu, Shiga 520-2192 Japan; 2 Molecular Neuroscience Research Center, Shiga University of Medical Science, Otsu, Shiga 520-2192 Japan; 3 Department of Neurology, Kyoto University Graduate School of Medicine, Kyoto, 606-8501 Japan; 4 Research Center for Animal Life Science, Shiga University of Medical Science, Otsu, Shiga 520-2192 Japan; 5 Institute for the Advanced Study of Human Biology (WPI-ASHBi), Kyoto University, Kyoto, 606-8501 Japan; 6 Department of Integrative Physiology, Shiga University of Medical Science, Otsu, Shiga 520-2192 Japan; 7 Division of Neurological Gene Therapy, Center for Open Innovation, Jichi Medical University, Tochigi, 320-0498 Japan; 8 Center for Gene and Cell Therapy, The Institute of Medical Science, The University of Tokyo, Tokyo, 108-0071 Japan; 9 Department of Integrative Anatomy, Graduate School of Medical Sciences, Nagoya City University, Nagoya, 467-8601 Japan

**Keywords:** Chemical biology, Neuroscience, Neurological disorders, Mechanisms of disease, Protein folding

## Abstract

Genetic mutations in fused in sarcoma (FUS) cause amyotrophic lateral sclerosis (ALS). Although mitochondrial dysfunction and stress granule have been crucially implicated in FUS proteinopathy, the molecular basis remains unclear. Here, we show that DHX30, a component of mitochondrial RNA granules required for mitochondrial ribosome assembly, interacts with FUS, and plays a crucial role in ALS-FUS. WT FUS did not affect mitochondrial localization of DHX30, but the mutant FUS lowered the signal of mitochondrial DHX30 and promoted the colocalization of cytosolic FUS aggregates and stress granule markers. The immunohistochemistry of the spinal cord from an ALS-FUS patient also confirmed the colocalization, and the immunoelectron microscope demonstrated decreased mitochondrial DHX30 signal in the spinal motor neurons. Subcellular fractionation by the detergent-solubility and density-gradient ultracentrifugation revealed that mutant FUS also promoted cytosolic mislocalization of DHX30 and aggregate formation. Interestingly, the mutant FUS disrupted the DHX30 conformation with aberrant disulfide formation, leading to impaired mitochondrial translation. Moreover, blue-native gel electrophoresis revealed an OXPHOS assembly defect caused by the FUS mutant, which was similar to that caused by DHX30 knockdown. Collectively, our study proposes DHX30 as a pivotal molecule in which disulfide-mediated conformational change mediates mitochondrial dysfunction and cytosolic aggregate formation in ALS-FUS.

## Introduction

ALS is a fatal motor neuron disease characterized by the degeneration of the upper and lower motor neurons. Most patients are diagnosed with sporadic ALS (SALS), while approximately only 10% of the patients are diagnosed with familial ALS (FALS). *FUS* gene mutations have been identified as a genetic cause of both SALS and FALS^1,2^. FUS is an RNA/DNA-binding protein associated with various cellular functions^3^. Most *FUS* mutations cluster in the low complexity domain (LCD) in the N-terminal, the second RGG domain, and the nuclear localization signal (NLS) in the C-terminal^4^. Although most ALS patients harboring FUS mutations show the classic ALS phenotype, several mutations located in the C-terminal, especially within the NLS, cause juvenile-onset sporadic ALS with rapid disease progression^5^.

The pathology of ALS-FUS is characterized by cytosolic aggregation of FUS proteins^1,6^. Recently, liquid–liquid phase separation (LLPS) has been implicated in protein aggregation. LLPS is a mechanism for the condensation of RNA molecules and RNA binding proteins (RBPs), resulting in the formation of dynamic membraneless compartments, such as stress granules (SGs) and P-granules^7,8^. FUS can form liquid compartments via the prion-like LCD and are recruited to SGs, while FUS mutants induce an aberrant phase transition into irreversible fibrillar hydrogels, which might be the molecular basis of FUS aggregation^9–11^. Although the increase in toxic properties conferred by ALS-FUS mutations has been supported by previous findings^12,13^, and many studies focusing on SGs and cytosolic aggregation have been reported^14–17^, the associated pathogenic mechanisms remain elusive.

Structures resembling cytosolic RNA granules have been identified close to mitochondrial nucleoids within the mitochondrial matrix^18,19^. Mitochondrial dysfunction is one of the major pathways involved in ALS pathogenesis^20–23^. For instance, mutations in the *CHCHD10* gene encoding mitochondrial proteins have been identified in frontotemporal dementia-ALS (FTD-ALS), which supports the importance of mitochondrial defects in motor neuron degeneration^24^. FUS also localizes in mitochondria, which can cause mitochondrial fragmentation and dysfunction along with defects in the axonal transport of mitochondria under pathological conditions^6,25–27^. Furthermore, reducing mitochondrially localized FUS via downregulation of HSP60 partially rescued mitochondrial defects and neurodegenerative phenotypes caused by the overexpression of FUS in vivo^26^. However, the mechanism by which FUS mutants induce mitochondrial dysfunction is unclear.

We identified DHX30, an ATP-dependent RNA helicase, as a FUS-interacting protein. DHX30 localizes mainly within the mitochondrial matrix, serves as a component of the mitochondrial RNA granule, and is required for mitochondrial ribosome assembly^28,29^. Our study revealed that FUS mutants induced both structural and functional defects in DHX30, leading to mitochondrial dysfunction and cytotoxicity, and recruited DHX30 to the cytosolic aggregation of FUS mutants. DHX30 may be crucially involved in neurodegeneration in ALS-FUS.

## Results

### Interactome analysis of FUS-interacting proteins

**Figure 1:**
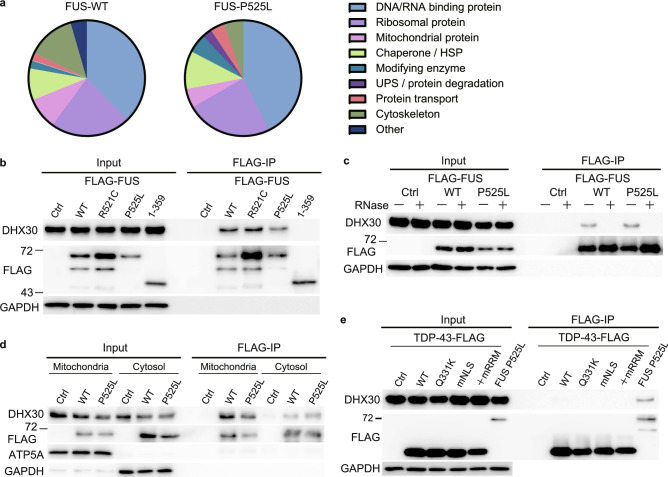
DHX30 interacts with both WT and mutant FUS in an RNA-dependent manner. (**a**) Immunoprecipitation was performed using FLAG antibody with SHSY5Y cells transfected with FLAG-tagged FUS WT, P525 L, 1–359, or the control vector. Purified protein complexes were confirmed by silver staining and then identified using mass spectrometry. Pie chart showing the categorization of the identified proteins interacting with FUS WT or P525 L. (**b**) Western blotting of input and IP samples with the indicated antibodies following FLAG-IP using HEK293A cells transfected with FLAG-FUS WT, R521C, P525L, 1–359 or control vector. GAPDH was used as the loading control. (**c**) Western blotting following FLAG-IP using cell lysates treated with RNase. (**d**) Western blotting following FLAG-IP using mitochondrial and cytosolic compartments. ATP5A and GAPDH were used as loading controls for the mitochondrial and cytosolic compartments, respectively. (**e**) FLAG-IP using HEK293A cells transfected with WT, ALS-associated mutations, cytoplasmic (mNLS), or aggregated (mNLS and mRRM) TDP-43-FLAG, FLAG-FUS P525L, or control vector.

To identify proteins that interact with FUS, FLAG-tagged constructs expressed under the cytomegalovirus promoter were generated for FUS wild-type (WT), a common ALS-associated mutant variant (P525L), and a deletion mutant devoid of the C′-terminus (1–359). Although FUS WT localizes mainly in the nucleus, the substitution of proline to leucine mutation (P525L) in NLS results in its mislocalization to the cytoplasm and causes a severe form of ALS^30,31^. FUS 1–359, which lacks NLS and a region responsible for RNA recognition and binding, was also detected in the cytoplasm, and the transgenic mice harboring this mutation showed FUS proteinopathy and severe motor phenotype^32^. To identify their binding partners, lysates from the human neuroblastoma cell line (SH-SY5Y), transiently transfected with these constructs by electroporation, were immunoprecipitated with FLAG-FUS. Western blotting of the inputs showed that the expression of FLAG-FUS was equivalent to endogenous FUS (Supplementary Fig. S1a). The immunoprecipitates were analyzed by silver staining to confirm the pull-down efficiency (Supplementary Fig. S1b). Using mass spectrometry analysis, we identified 45, 75, and 8 proteins enriched in the FLAG-FUS WT, P525L, and 1–359 immunoprecipitates, respectively, relative to the control (Supplementary Table S1). Based on the result of FUS 1–359, the RNA-recognition motif is also considered to be critical for protein association. On comparing the FUS WT and P525L interacting proteins, 28 common proteins were found, and there was no major difference in the proportion of their functions. Consistent with these results, RNA-binding ribonucleoproteins, splicing factors, and ribosomal proteins were predominantly observed (Fig. 1a).

### FUS associates with DHX30 in an RNA-depending manner

Among FUS-interacting proteins, we focused on DHX30, which is an RNA helicase required for mitochondrial ribosome biogenesis and has also been reported to cause neurodevelopmental disorders^29,33^. First, we investigated the subcellular localization of DHX30 in HEK293A cells overexpressing EGFP-tagged FUS and mCherry-tagged DHX30-WT using immunofluorescence (IF). IF assay revealed strong colocalization of GFP-FUS P525L with mCherry-DHX30 in the cytoplasm and mild colocalization of GFP-FUS WT or P525L with mCherry-DHX30 puncta in the nucleus (Supplementary Fig. S2a). DHX30 expressed exogenously increased the aggregate formation of FUS P525L compared to other interacting proteins identified in this study (Supplementary Fig. S2b). Knockdown of DHX30 showed the potential to reduce the size of the aggregates but significantly increased their number (Supplementary Fig. S2c). These results suggested that DHX30 enhances FUS aggregate formation, but depletion of DHX30, which is critical for mitochondrial protein synthesis, may also increase the aggregate formation via mitochondrial impairment.

Next, we investigated the interaction between FUS and DHX30 in HEK293A cells expressing FLAG-FUS WT, P525L, 1–359, or R521C, which is the most common ALS-associated mutant variant in NLS. An immunoprecipitation assay using FLAG-FUS confirmed that both FUS WT and the ALS causative mutants interacted with endogenous DHX30, except for the 1–359 lacking major RNA binding motifs (Fig. 1b). RNase A treatment of the cell lysates decreased the interaction of DHX30 with either FUS WT or P525L (Fig. 1c). An immunoprecipitation assay using FLAG-FUS after subcellular fractionation showed that either FUS WT or P525L was associated with DHX30 in the mitochondrial and cytosolic fractions (Fig. 1d). To test whether DHX30 interacts with TAR DNA-binding protein 43 (TDP-43), another ALS causative and RNA-binding protein, we performed an immunoprecipitation assay using FLAG antibody in HEK293A overexpressing TDP-43-FLAG WT, a familial ALS-linked mutant variant (Q331K), a cytoplasmic mislocalized artificial mutant (mNLS: R82L/K83Q), or an intracytosolic aggregate-forming mutant (mNLS/mRRM: C173S/C175S)^34^. Neither TDP-43 WT nor the mutants co-immunoprecipitate with DHX30 (Fig. 1e). Collectively, these data indicate that DHX30 binds to FUS WT and P525L in an RNA-dependent manner.

### DHX30 colocalized with FUS cytosolic aggregates, and the DHX30 signals in mitochondria are diminished in human cultured cells and spinal motor neurons of an ALS patient with the FUS P525L mutation

**Figure 2:**
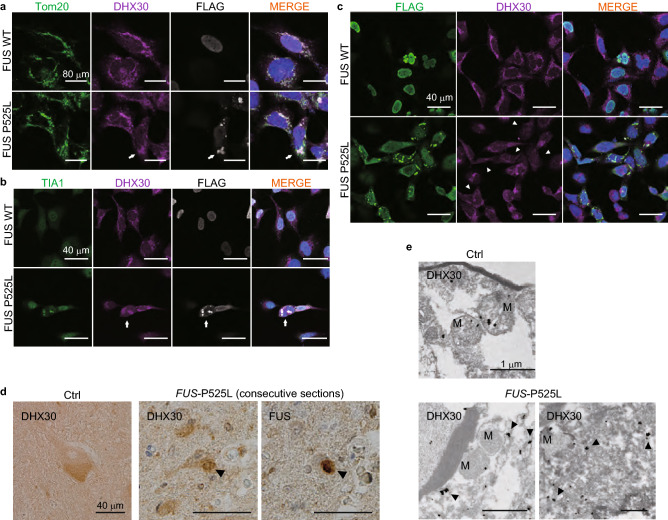
DHX30 colocalized with FUS cytosolic aggregates, and the DHX30 signals in mitochondria are diminished in human cultured cells and spinal motor neurons of an ALS patient with the FUS P525L mutation. (**a**–**c**) Immunocytochemistry of HEK293A cells transfected with FLAG-FUS WT or P525L. DHX30 localized mainly within mitochondria, positive for Tom20, in the cells expressing FUS WT (**a**). Colocalization of DHX30 and FUS cytosolic aggregates, indicated by the white arrows, was shown in the cells expressing FUS P525L, which were negative for Tom20 and positive for TIA-1, a SGs marker (**a**, **b**). The fluorescence intensity of DHX30 within mitochondria was decreased in the cells expressing FUS P525L, indicated by the white arrowheads (**c**). (**d**) Representative images of immunohistochemistry using DAB stain for FUS and DHX30 in the spinal motor neurons of the ALS patient with a FUS P525L mutation and a patient with brain infarction as control. The consecutive sections show colocalization of DHX30 and FUS aggregates. (**e**) Immunogold electron micrographs for DHX30 from the spinal motor neurons of ALS-FUS show that the signal of DHX30 is decreased in mitochondria and increased in the cytosol. M presents mitochondria, and black arrows indicate the signal of DHX30 in the cytosol.

To examine whether FUS mutants affect the subcellular localization of endogenous DHX30, we performed an IF assay using HEK 293A cells transfected with FLAG-FUS-WT or P525L. In the majority of cells expressing FUS WT, DHX30 localized within mitochondria, which were positive for Tom20, a mitochondrial marker (Fig. 2a). It is consistent with the mitochondrial distribution of DHX30 reported previously^28^. In cells expressing FUS P525L, DHX30 colocalized with the cytosolic aggregates of FUS P525L to some extent (Fig. 2a). The aggregates were positive for TIA1, a well-established SGs marker (Fig. 2b). However, the most common finding in the cells was a reduced signal of DHX30 within mitochondria, in contrast to FUS WT showing granular localization of DHX30 (Fig. 2a, c). We also performed immunoelectron microscopy to evaluate the localization of FUS to mitochondria in HEK293A cells expressing FLAG-FUS or Ctrl (Supplementary Fig. S3). Immunogold electron micrographs for FUS showed that both endogenous and exogenous FUS proteins were partially localized to mitochondria, and the overexpressed mutant showed an increased level of mitochondrial localization, as previously reported^26^. These images were consistent with the immunoprecipitation assay in which FUS and DHX30 interacted in the cytosolic and mitochondrial fraction (Fig. 1d).

To investigate the localization of DHX30 in motor neurons of ALS-FUS patients, we performed immunohistochemistry using consecutive sections within the anterior horn of the lumbar spinal cord from an ALS-FUS patient with FUS P525L mutation and a control patient with cerebral infarction. We found that FUS aggregates frequently colocalized with FUS to varying degrees in an ALS-FUS patient (Fig. 2d, Supplementary Fig. S4). We also performed immunoelectron microscopy to evaluate the localization of DHX30 in the mitochondria of ALS-FUS (Fig. 2e). The DHX30 signal in the mitochondria of the spinal motor neurons of the ALS-FUS patient was decreased, which was increased in the cytosol. Considering that DHX30 significantly increased FUS aggregates (Supplementary Fig. S2b), these imaging data suggested that DHX30 might promote FUS aggregate formation in the cytosol, and the loss of function of DHX30 occurred in mitochondria in ALS-FUS.

### FUS mutants do not affect the expression level and subcellular localization of DHX30 but mildly recruit DHX30 to FUS aggregates

**Figure 3:**
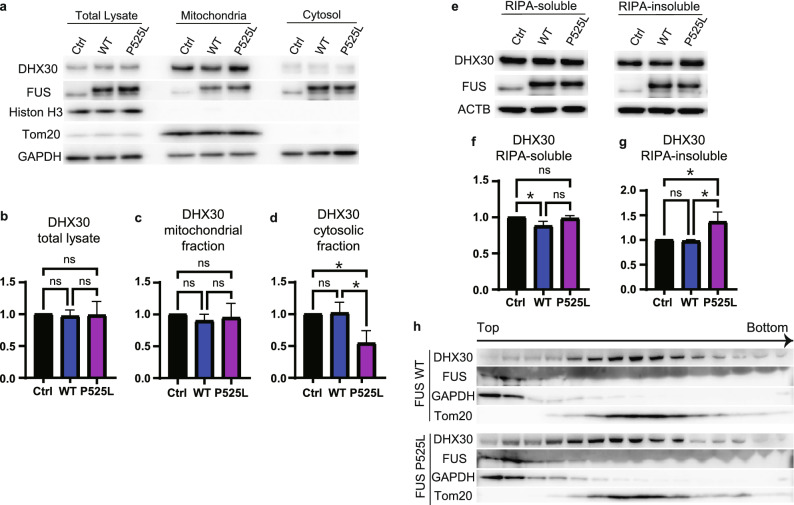
FUS mutants do not affect the expression level and subcellular localization of DHX30, except for the mild recruitment of DHX30 to FUS cytosolic aggregates. (**a**) Western blotting following a subcellular fractionation by differential centrifugation using cells expressing FLAG-FUS WT, P525L, or Ctrl at 48 h. (**b**-**d**) Quantitative analysis of DHX30 levels shown in (**a**). Each data point was obtained by normalization to GAPDH (**b** and **d**) and Tom20 (**c**) and comparison with control. n = 3 independent experiments. (**e**) Western blotting for detergent insolubility of DHX30. (**f**, **g**) Quantitative analysis of DHX30 levels shown in (**e**). Each data point was obtained by normalization to ACTB and comparison with control. n = 3 independent experiments. (**h**) Western blotting following ultracentrifugation in Percoll gradients. The slight shift of DHX30 was observed in cells expressing FUS P525L. Data were analyzed using a one-way ANOVA with post hoc Tukey’s multiple comparison tests (*p < 0.05, ns = not significant). Error bars represent mean ± SEM.

To test whether this observation indicates the altered subcellular localization of DHX30 by FUS P525L, we performed subcellular fractionation by differential centrifugation, followed by Western blotting (Fig. 3a). Contrary to our expectations, the expression level of DHX30 in both the total lysate and the mitochondrial fraction was unchanged with or without the FUS mutant (Fig. 3b,c). Although cytosolic DHX30 appeared decreased (Fig. 3d), this fractionation protocol might not correctly estimate the effect of cytosolic aggregates. We then separated cell lysates into RIPA-soluble or -insoluble fractions to determine whether FUS P525L affects the detergent solubility of DHX30 (Fig. 3e). Western blotting analysis revealed that FUS P525L increased the detergent insolubility of DHX30, suggesting an unfolding state of DHX30, which was not the case in FUS WT (Fig. 3f,g). Further, we investigated the post-nuclear fraction using density-gradient ultracentrifugation in Percoll (Fig. 3h). In cells expressing either FUS WT or P525L, DHX30 was mainly localized within the mitochondrial fraction. However, compared to FUS WT, a mild shift of DHX30 to FUS-enriched fraction was observed in cells expressing P525L, which implies colocalization with DHX30 and FUS aggregation. Collectively, these data indicate that the subcellular localization of DHX30 was not affected by the expression of FUS WT and mutants, except for the small amount of DHX30 recruited to FUS aggregates.

### FUS mutants affect the structure of DHX30

**Figure 4:**
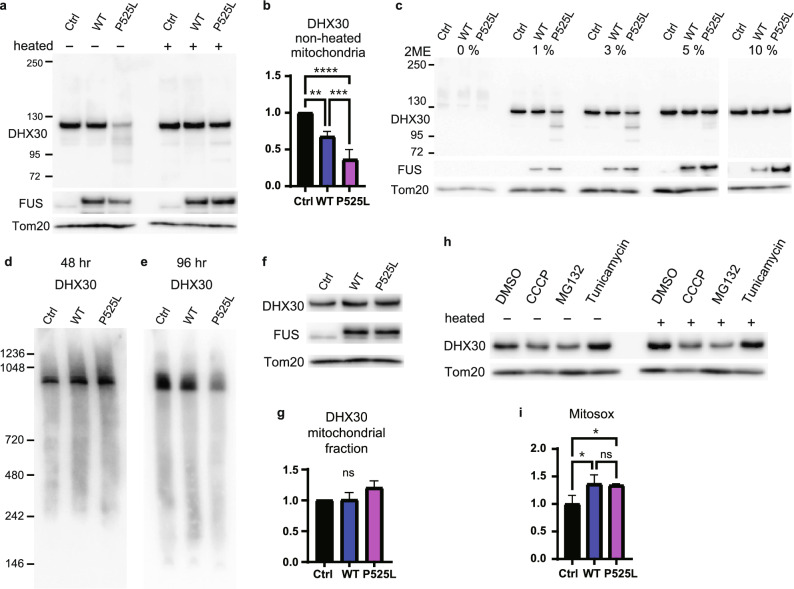
FUS mutants affect the structure of DHX30. (**a**) Western blotting of the non-heated or heated mitochondrial lysates of HEK293A cells transfected with FLAG-FUS WT, P525L, or control vector at 48 h. (**b**) Quantitative analysis of DHX30 levels shown in (**a**). Each data point was obtained by normalization to Tom20 and comparison with control. n = 3 independent experiments. (**c**) Western blotting of the mitochondrial lysates of cells expressing FLAG-FUS WT, P525L, or Ctrl treated with the various concentration of 2-ME at 48 h. (**d**, **e**) BN-PAGE analysis with the DHX antibody of the mitochondrial lysates of cells expressing FLAG-FUS WT, P525L, or Ctrl at 48 h (**d**) and 96 h (**e**). (**f**) Western blotting with the indicated antibodies of mitochondrial lysates from HEK293A cells expressing FLAG-FUS WT, P525 L, or control vector at 96 h. (**g**) Quantitative analysis of DHX30 levels shown in (**f**). Each data point was obtained by normalization to Tom20 and comparison with control. n = 3 independent experiments. (**h**) Western blotting of the non-heated or heated mitochondrial lysates of cells treated with DMSO 0.1%, CCCP 10 μM, MG132 10 μM, or Tunicamycin 1 μg/ml for 12 h. (**i**) Quantification of Mitosox signal intensity of expressing FLAG-FUS WT, P525L, or Ctrl at 48 h. Each data point was obtained by comparison with the control. n = 3 independent experiments. Data were analyzed using a one-way ANOVA with post hoc Tukey’s multiple comparison tests (*p < 0.05, **p < 0.01, ***p < 0.005 and ****p < 0.001, ns = not significant). Error bars represent mean ± SEM.

Despite similar expression levels of DHX30 in mitochondria of WT and P525L FUS, cells expressing FUS mutants showed reduced immunoreactivity against the anti-DHX30 antibody in IF analysis, which was the same as in spinal motor neurons of ALS-FUS by the immunoelectron microscope (Fig. 2c,e). Consequently, we hypothesized that the conformation of mitochondrial DHX30 was altered following its interaction with FUS P525L. Thus, we performed Western blotting using the mitochondrial fractions under various denaturing conditions. First, the mitochondrial lysates from HEK293A cells transfected with FLAG-FUS WT or P525L were treated with 5% 2-mercaptoethanol (2ME) with or without heating. We found different migration patterns of DHX30 with FUS P525L under non-heating conditions compared to that of the vector or WT FUS (Fig. 4a). In the presence of FUS P525L, the main signal of DHX30 at approximately 130 kDa without heating, decreased drastically, accompanied by the presence of lower molecular species. The WT sample also showed a similar but mild trend (Fig. 4b). These signal changes were recovered by heating. It was suggested that the conformation of DHX30 was affected by the expression of FUS, especially P525L. Next, to examine whether aberrant disulfide interaction is involved in the conformational change of DHX30, we evaluated the effect of different concentrations of 2ME with mild heating at 37 °C for 5 min on the migration of DHX30 by Western blotting. The results show that the DHX30 signal under the presence of FUS P525L decreased prominently in the sample at low concentrations of 2ME, and that of the WT sample also decreased mildly. The signals recovered upon increasing the concentrations of 2ME and were completely recovered in the sample using 10% 2ME (Fig. 4c). These results indicated that FUS P525L did not affect the expression level of DHX30 but induced structural alterations mediated by aberrant disulfide bonds. Subsequently, to investigate whether the structural alteration affects protein complexes containing DHX30, mitochondria isolated from cells expressing FUS WT or P525L were separated by blue native PAGE (BN-PAGE). DHX30 immunoblotting showed a decrease in DHX30 levels at 96 h after transfection, but not at 48 h (Fig. 4d,e). The DHX30 levels were comparable at both times in the denaturing Western blotting experiment (Figs. 3a,c, 4f,g). These results indicate that the protein complex containing DHX30 was structurally affected in a time-dependent manner in the presence of FUS, especially P525L.

The mechanism by which FUS P525L causes a disulfide-mediated conformational change in DHX30 is unclear. To examine whether the conformational alteration is due to mitochondrial dysfunction or other proteotoxic stress, we tested carbonyl cyanide m-chlorophenyl hydrazone (CCCP), which causes depolarization of mitochondria, MG132, a proteasome inhibitor, and Tunicamycin, a protein glycosylation inhibitor inducing endoplasmic reticulum (ER) stress and unfolded protein response. Resultantly, the structural change of DHX30 was not observed in response to the stresses, though CCCP and MG132 decreased the expression of DHX30 in mitochondria (Fig. 4h). Oxidative stress is also a possible pathomechanism in mitochondria, in addition to the direct interaction between FUS and DHX30. Therefore, we evaluated the levels of mitochondrial superoxide in cells expressing FUS WT or P525L using MitoSOX (Fig. 4i). Superoxide production increased comparably in both FUS WT and P525L. These results suggest that mitochondrial dysfunction and proteotoxic or oxidative stress are not significant causes of the structural alterations in DHX30. Thus, it is conceivable that FUS mutants induce structural alteration of DHX30 through the interaction between FUS and DHX30.

### FUS mutants decrease the expression levels of the mitochondrially encoded proteins and induce mitochondrial dysfunction and cytotoxicity, suggesting the loss of function of DHX30

**Figure 5:**
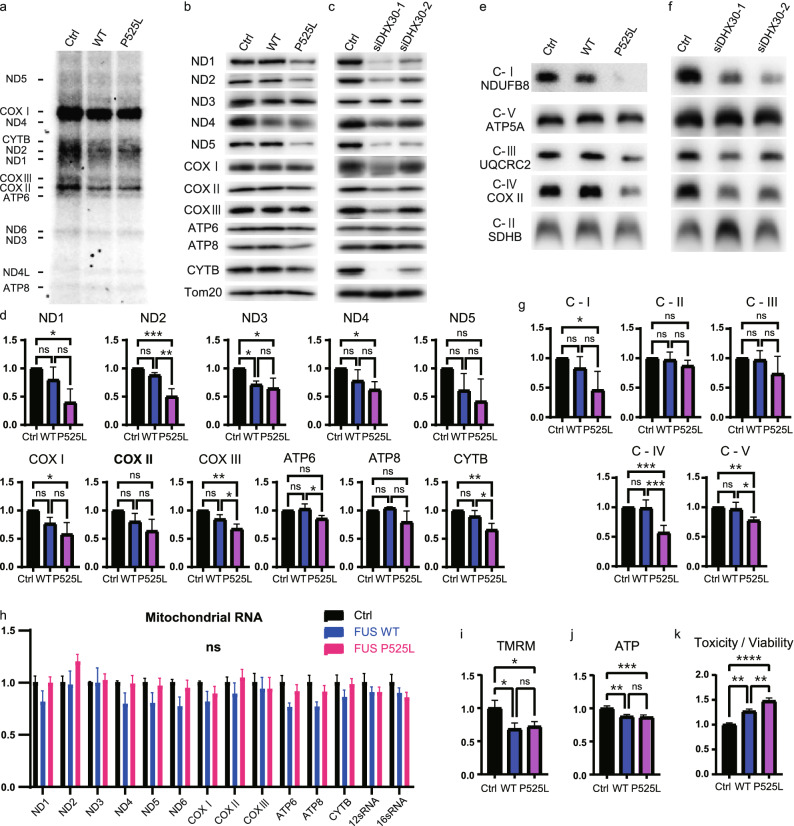
FUS mutants decrease the expression level of the mitochondrially encoded protein and induce mitochondrial dysfunction and cytotoxicity, suggesting the loss of function of DHX30. (**a**) Pulse-labeling translation experiment of the 13 mitochondrially encoded polypeptides (seven subunits of complex I [ND], three subunits of complex IV [COX], two subunits of complex V [ATP], and one subunit of complex III [CYTB]) in HEK293A cells expressing FLAG-FUS WT, P525L, or Ctrl at 48 h. (**b**, **c**) Western blotting with the indicated antibodies of the mitochondrial lysates of cells transfected with FLAG-FUS WT, P525L, Ctrl (**b**), siRNA-DHX30, or Ctrl (**c**) at 96 h. (**d**) Quantitative analysis of the protein levels shown in (**b**). Each data point was obtained by normalization to Tom20 and comparison with control. n = 3 independent experiments. (e–f) BN-PAGE analysis of cells expressing FUS P525L (**e**) or siRNA-mediated knockdown of DHX30 (**f**) at 96 h shows an OXPHOS defect as revealed by subunit-specific antibodies against individual OXPHOS complexes. (**g**) Quantitative analysis of the band intensities shown in (**e**). Each data point was obtained by comparison with the control. n = 4 independent experiments. (**h**) Quantitative real-time PCR analysis of the mitochondrially encoded gene expression of cells expressing FLAG-FUS WT or P525L relative to that of Ctrl at 48 h. Each result was normalized to ACTB. n = 3 independent experiments. (**i**–**k**) Quantification of TMRM (tetramethylrhodamine methyl ester) (**i**) and ATP (**j**) signal intensity and R110/AFC fluorescence ratio (**k**) of expressing FLAG-FUS WT, P525L, or Ctrl at 96 h. Each data point was obtained by comparison with the control. n = 3 independent experiments. Data were analyzed using a one-way ANOVA with post hoc Tukey’s multiple comparison tests (*p < 0.05, **p < 0.01, ***p < 0.005 and ****p < 0.001, ns = not significant). Error bars represent mean ± SEM.

In a previous report, DHX30 was shown to be required for mitochondrial ribosome biogenesis, and the depletion of DHX30 resulted in impairment of mitochondrial protein synthesis and an OXPHOS assembly defect^29^. To investigate whether the FUS-mediated conformational change of DHX30 may affect the functions of DHX30, we pulse-labeled the mitochondrial translation products with a mixture of [^35^S]-methionine/cysteine in the presence of emetine, a cytosolic translation inhibitor, using HEK293A cells transfected with FLAG-FUS WT or P525L at 48 h. We observed an overall decrease in mitochondrial protein synthesis in FUS WT and P525L cells (Fig. 5a). The synthesis of ND1, ND2, COX I, COX II, and COX III polypeptides was markedly affected. To evaluate the protein levels, we performed Western blotting of the mitochondrial lysates from HEK293A cells transfected with FLAG-FUS WT, P525L, or siRNA-DHX30 at 96 h (Fig. 5b, c). We observed an overall decrease in the protein levels of the mitochondrially encoded proteins in cells transfected with FUS, which was more prominent in FUS P525L than in WT (Fig. 5d). In siRNA-DHX30-treated cells, similar mitochondrial protein reduction profiles were observed. To test whether the decrease in the mitochondrially encoded proteins resulted in an OXPHOS assembly defect, we performed BN-PAGE of the mitochondrial lysate at 96 h after transfection (Fig. 5e,f). In cells expressing FUS P525L, we observed a severe OXPHOS assembly defect in complex I and IV and a mild defect in complex V (Fig. 5g). A similar defect, except for that in complex V, was observed in DHX30-depleted cells, and no significant decrease was observed in cells expressing FUS WT. These data indicate the possibility that the expression of FUS P525L might lead to the loss of function of DHX30 by conformational alteration. As for complex V, a different mechanism might be responsible. For instance, a previous report documented that FUS can interact with the catalytic subunit of mitochondrial ATP synthase (ATP5B), leading to a defect of complex V^35^. To test whether the decrease in the expression of the mitochondrially encoded proteins was caused by impaired mitochondrial transcription, we performed a quantitative RT-PCR assay in cells expressing FUS WT or P525L at 48 h. We found no significant change in the amount of mRNA encoded in mitochondrial DNA (Fig. 5h).

To investigate whether the expression of FUS impairs mitochondrial function, we evaluated mitochondrial membrane potential and ATP production in cells expressing FUS WT or P525L at 96 h. Notably, either FUS WT or P525L impaired both functions (Fig. 5i,j). It is conceivable that the higher expression level of FUS WT is also toxic, which is supported by a report describing several mutations in the 3′ untranslated region (UTR) of the *FUS* gene identified in ALS patients^36^. We also confirmed the cytotoxicity of FUS using fluorescence/luminescent analysis (Multitox assay) in HEK293A cells, in which FUS P525L was shown to be more toxic (Fig. 5k).

These results indicate that FUS mutants impaired mitochondrial translation and caused OXPHOS assembly defects, leading to mitochondrial dysfunction and cytotoxicity. Since DHX30-depleted cells displayed similar results, it is suggested that the P525L FUS-mediated structural alteration of DHX30 is linked to the loss of function of DHX30.

### FUS mutants induce the structural alteration of DHX30 in SH-SY5Y cells

The expression levels of FUS WT or P525L in HEK293A cells were higher than in physiological conditions. We thus established SH-SY5Y cells conditionally and stably expressing FUS WT and P525L protein by tetracycline. We adopted the TET-ON 3G inducible expression system and the PiggyBac transposon vector system, in which pPB-flox(CAG-Tet3G-IN; TRE3G-cHA-pA), pPB-flox(CAG-Tet3G-IN; TRE3G-FLAG-FUS WT-pA), and pPB-flox(CAG-Tet3G-IN; TRE3G-FLAG-FUS P525L-pA) were used.

**Figure 6:**
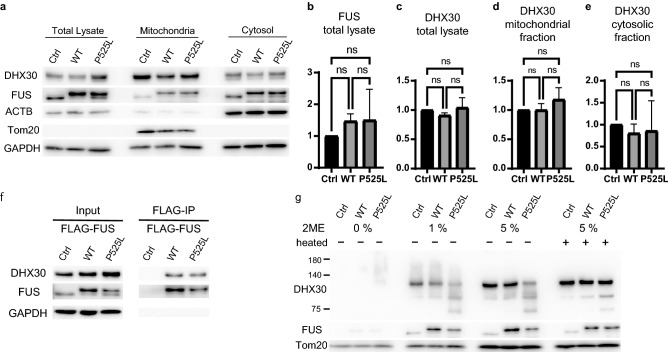
FUS mutants induce the structural alteration of DHX30 in SHSY-5Y cells. (**a**) Western blotting following a subcellular fractionation by differential centrifugation using SH-SY5Y cells with stable expression of FLAG-FUS WT, P525L, or Ctrl (144 h post-induction). (**b**–**e**) Quantitative analysis of FUS or DHX30 levels shown in (**a**). Each data point was obtained by normalization to GAPDH (**b**, **c**, **e**) and Tom20 (**d**) and comparison with control. n = 3 independent experiments. (**f**) Western blotting of input and IP samples with the indicated antibodies following FLAG-IP using SH-SY5Y cells (48 h post-induction). GAPDH was used as a loading control. (**g**) Western blotting of the mitochondrial lysates of SH-SY5Y cells (72 h post-induction) treated with various concentrations of 2-ME or heating. Data were analyzed using a one-way ANOVA with post hoc Tukey’s multiple comparison tests (ns = not significant). Error bars represent mean ± SEM.

We next attempted to investigate whether FUS mutants could affect the subcellular localization of DHX30. The lysates from SHSY-5Y cells at 144 h after induction were subjected to subcellular fractionation by differential centrifugation and were analyzed by Western blotting (Fig. 6a). We confirmed that the expression levels of FUS WT or P525L were comparable to that of endogenous FUS (Fig. 6b). Neither FUS WT nor P525L affected the expression levels of DHX30 in the total lysate, mitochondrial or cytosolic fractions (Fig. 6c-e). Next, we investigated the interaction between FUS and DHX30 in SHSY-5Y cells at 48 h after induction (Fig. 6f). An immunoprecipitation assay using FLAG-FUS confirmed that both FUS WT and P525L interacted with DHX30 even at expression levels comparable to endogenous. The conformational change in DHX30 was also analyzed by Western blotting of mitochondrial lysates from SH-SY5Y cells at 72 h after induction (Fig. 6g). The signal intensity of DHX30 in the FUS P525L sample prepared with 1% or 5% 2ME and mild heating decreased drastically and showed some extra signals at lower molecular weights. The WT sample also showed a similar but mild trend. These signal changes were recovered and migrated to a single basic band upon heating. Since human neuronal cells (SHSY-5Y) recapitulated those in HEK293 cells, it is plausible that the interaction between FUS and DHX30 is general irrespective of cell type. It was also confirmed that the conformational change in DHX30 induced by FUS is not an artificial phenomenon since the expression levels of FUS were comparable to the physiological state.

### Overexpression of DHX30 failed to rescue mutant FUS-induced mitochondrial damage and cytotoxicity

To investigate whether co-expression of DHX30 could rescue FUS toxicity, we investigated HEK293A cells expressing FUS P525L, co-expressing DHX30 WT, or control vector at 96 h. Disappointingly, co-expressing of DHX30 failed to prevent cytotoxicity of FUS P525L using Multitox assay (Supplementary Fig. S5a). Further, BN-PAGE analysis showed that the protein complexes containing DHX30 and the OXPHOS assembly defected by FUS P525L were not recovered by the co-expression of DHX30 (Supplementary Fig. S5b,c).

To examine whether overexpression of DHX30 itself could cause mitochondrial damage, we generated HEK293A cells stably expressing DHX30 WT and R493H protein in a tetracycline-dependent manner, using pPB-flox(CAG-Tet3G-IN; TRE3G-cHA-pA), pPB-flox(CAG-Tet3G-IN; TRE3G-DHX30 WT-Myc-pA), and pPB-flox(CAG-Tet3G-IN; TRE3G-DHX30 R493H-Myc-pA) using the TET-ON 3G inducible expression system and the PiggyBac transposon vector system. R493H has been reported as a causative mutation of neurodevelopmental disorder and to be more prone to aggregate formation than WT^33^. The structural change of DHX30 and OXPHOS assembly defects were observed in cells expressing either DHX30 WT or R493H at 96 h after induction, more prominently in R493H (Supplementary Fig. S5d,e). Considering that DHX30 could promote the formation of FUS aggregates (Supplementary Fig. S2b), this rescue method has a limitation not enough to evaluate the involvement of DHX30 in FUS toxicity because overexpression of DHX30 WT exacerbates the structural defects of FUS P525L and DHX30. Altogether, these findings suggest that the loss of function of DHX30 might underlie the molecular basis of mitochondrial dysfunction in ALS-FUS, and DHX30 may play a role in FUS aggregate formation.

## Discussion

We identified DHX30 as a FUS-interacting protein via immunoprecipitation and mass spectrometry analyses of SHSY-5Y cells. DHX30 is an ATP-dependent RNA helicase located mainly within the mitochondrial matrix and comprises the mitochondrial RNA granule^28,29^. FUS interacted with DHX30 in both mitochondria and cytosol in an RNA-dependent manner, and FUS mutants disrupted the conformation of mitochondrial DHX30 via excessive disulfide formation. We propose that DHX30 is a key molecule underlying mitochondrial dysfunction in ALS-FUS.

The exact mechanism by which FUS mutants caused the structural alteration of mitochondrial DHX30 remains unclear. One plausible hypothesis is aberrant phase transitions of FUS and DHX30. FUS is an RBP with intrinsically disordered, prion-like LCD that has properties of LLPS and forms SGs. ALS causative mutations in FUS alter these properties, leading to aberrant phase transitions into irreversible fibrillar hydrogels that sequester RNP cargo^10,11^. In FUS-ALS knock-in mice, FUS mutants enhanced not only its own insolubility but also that of related RBPs^37^. DHX30 is also an RBP predicted to have intrinsically disordered regions in UniProt, comprises the mitochondrial RNA granule^28,29^, and accumulates in SGs^33^, all of which implies the involvement of DHX30 in LLPS properties. From the above, it is hypothesized that the interaction between FUS mutants and DHX30 in mitochondria may lead to aberrant phase transition and structural alteration of DHX30, though further study is needed.

From the IF assay of HEK293A cells expressing FUS mutants, it is difficult to deny that mitochondrial DHX30 might be reduced in some cells with FUS aggregates. However, a major reduction of mitochondrial DHX30 was not observed in the subcellular fractionation of HEK293A cells expressing FUS mutants, in which the transfection efficiency was high, and aggregates were observed in a relatively large number of the cells. Furthermore, no obvious localization changes of DHX30 were observed in SH-SY5Y cells with stable expression of FUS mutants. From these findings, it is plausible that, at least in cultured cells, the expression level of mitochondrial DHX30 itself is not altered. However, the DHX30 signal in the mitochondria was decreased in immunoelectron microscopy of the spinal motor neurons from the ALS-FUS patient, and mitochondrial DHX30 might decrease in situations where FUS aggregates are formed for a long period.

DHX30 is crucial for mitochondrial ribosome assembly. Its depletion leads to impaired mitochondrial translation and OXPHOS assembly defects in fibroblasts and causes early embryonic lethality in mice with homozygous deficiency of HelG/DHX30^29,38^. Our study demonstrated that the expression of FUS mutants impaired mitochondrial translation and protein synthesis, leading to the OXPHOS assembly defect, similar to the phenomenon observed in DHX30-depleted cells. These findings imply that the structural alteration of DHX30 induced by FUS mutants results in the loss of function of DHX30. The assembly of complexes I and IV is commonly disrupted in cells expressing FUS mutants and DHX30-depleted cells, whereas the assembly defect of Complex V was observed only in cells expressing FUS. This divergence is consistent with a previous report demonstrating that FUS can interact with ATP5B and disrupt the formation of complex V in human cultured cells^35^.

An immunohistochemical study of samples obtained from an ALS-FUS patient strongly supports the involvement of DHX30 in ALS-FUS pathogenesis. DHX30 colocalized with FUS in cytosolic aggregates, accompanied by the signal decrease of mitochondrial DHX30 in the spinal cord sections from a FUS-P525L ALS patient. An immunofluorescent study of human cultured cells expressing FUS mutants yielded similar results. These findings suggest that the loss of function of DHX30 plays a pivotal role in the pathogenesis of ALS-FUS. The missense mutations in the DHX30 gene have been identified as a genetic cause of neurodevelopmental disorders characterized by global developmental delay, intellectual disability, severe speech impairment, and gait abnormalities^33,39^. Although the role of DHX30 in ALS requires further studies, this genetic evidence supports our notion that FUS-related damage in DHX30 has a considerable impact as a potential mechanism of neurodegeneration.

Simultaneous overexpression of DHX30 onto FUS fails to rescue the mitochondrial defects and cytotoxicity by FUS mutants and is one of the limitations to endow the definite role of DHX30 that might argue against our view of the loss of functions of DHX30 in FUS proteinopathy. One plausible reason is that excessive DHX30 could deteriorate the structural defects of mitochondrial DHX30 and accelerate the aggregate formation of FUS mutants. The excess amount of DHX30 may impair the formation of its mitochondrial protein complexes. Moreover, our results indicated that DHX30 diffusely localized throughout the cytosol and increased FUS cytosolic aggregation in human cultured cells. Recent reports have demonstrated that DHX30 is also present in the cytosol^40,41^ and can colocalize with SGs^33,39^. DHX30 also inhibits p53-dependent apoptosis by controlling the translation of mRNAs acting via the 3′ UTR CGPD-motif^42^. These findings prompted us to reconsider the role of cytosolic DHX30 in ALS-FUS. Therefore, other approaches are necessary to obtain a rationale for DHX30 as a therapeutic target, such as small compounds, peptides, or nucleic acids that inhibit the interaction between FUS and DHX30.

On the other hand, regulation of FUS translocation into mitochondria is a therapeutic option. The Inhibition of the transfer of FUS to mitochondria partially rescued mitochondrial damage and neurodegenerative phenotypes in transgenic flies^26^. As for other RNA-binding proteins involved in most SALS and some FALS cases, TDP-43, characterized by aberrant redistribution into the cytoplasm and aggregate formation^43–45^, also causes mitochondrial dysfunction. The prevention of TDP-43 mitochondrial localization also ameliorates TDP-43-induced mitochondrial dysfunction and neuronal loss and improves the phenotypes of transgenic mutant TDP-43 mice^46^. Therefore, mitochondrial dysfunction has long been recognized as a potential therapeutic target. However, numerous human clinical trials targeting mitochondria have been unsuccessful^23^. Edaravone, an FDA-approved free radical scavenger, has exhibited only a small effect in reducing disease progression in early ALS patients^47^. Therefore, target molecules or pathways involved in mitochondrial dysfunction in ALS should be considered in the development of ALS therapies. Reductions in the activities of respiratory chain complexes have been reported in the post-mortem spinal cord of patients with SALS, and complex IV activity was reduced from the pre-symptomatic stage in the forebrain of transgenic mice harboring the G93A mutation in *superoxide dismutase 1* (*SOD1*), a causative mutation of ALS^48,49^. Therefore, mitochondrial respiration and ATP production have attracted considerable attention concerning motor neuron degeneration. Therefore, elucidating how RNA/DNA binding proteins accumulate in mitochondria and affect mitochondrial RNA granules is an immediate issue in understanding motor neuron degeneration in ALS. Our study proposes DHX30 as a novel therapeutic target against mitochondrial dysfunction in ALS.

There are several limitations other than the insufficient rescue effects. First, we examined only one autopsy case to confirm the colocalization between the FUS mutant and DHX30, which could not entirely exclude the variability. We did not use iPS cells because of their inaccessibility. However, intensive cell culture and cell studies, including immunoprecipitation and IF studies, provide substantial evidence to support our hypothesis. Second, the specificity of DHX30’s involvement in ALS-FUS remains elusive, especially for other RNA-binding proteins such as TDP-43. DHX30 was included as one of the interactomes with TDP-43^50^. However, our investigation demonstrated a much higher affinity for FUS than TDP-43 with DHX30. Nevertheless, it is intriguing to focus on DHX30 as pivotal machinery in ALS associated with RNA-interacting proteins.

**Figure 7:**
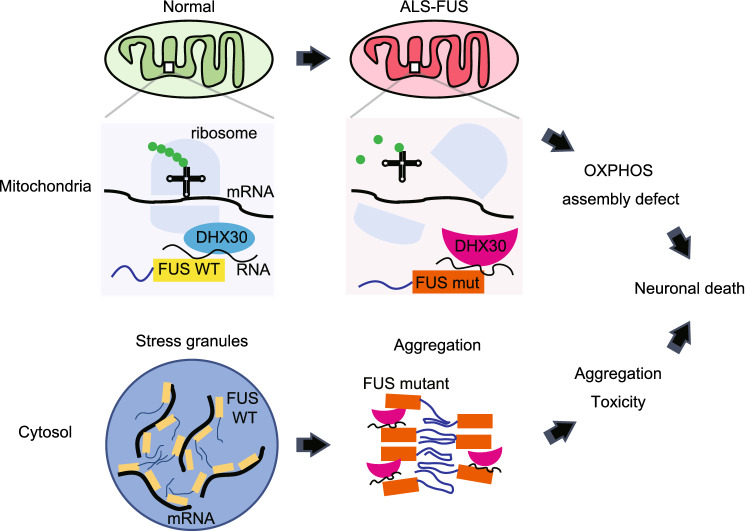
A schema of the pathophysiology of ALS*-*FUS associated with DHX30. We propose that DHX30, an ATP-dependent RNA helicase required for mitochondrial ribosome assembly, plays a pivotal role in the pathophysiology of ALS-FUS. FUS interacted with DHX30 in an RNA-dependent manner. FUS mutants could induce structural alterations of DHX30 mediated by aberrant disulfide bonds, leading to the loss of function of DHX30 in mitochondria, which resulted in the decreased expression levels of the mitochondrially encoded proteins, an OXPHOS assembly defect, and mitochondrial dysfunction. In the cytosol, FUS mutants could be recruited DHX30 to the site of its cytosolic aggregation, which might exacerbate the toxicity of aggregation.

In conclusion, we have shown that FUS interacts with DHX30, an RNA helicase localized mainly within mitochondria, in an RNA-dependent manner, and FUS mutants induced structural and functional defects in DHX30. This pathway might underlie mitochondrial dysfunction in ALS-FUS (Fig. 7). Further clarification of this pathway and appropriate intervention may open a new therapeutic window for managing ALS.

## Methods

### Cell culture, transfection, and generation of stable cell lines

All cultured cells were maintained at 37 °C under 5% CO_2_ and 100% humidity. HEK293A was maintained in high-glucose DMEM containing 10% fetal bovine serum (FBS). FuGene HD Transfection Reagent (Promega) was used for plasmid transfection. Stealth RNAi duplex constructs (Life Technologies) were used for the transient knockdown of DHX30 (HSS177019, HSS117877). Stealth siRNA duplexes were transiently transfected into cells using Lipofectamine RNAiMAX (Life Technologies), according to the manufacturer’s specifications. For the experiments at 96 h after transfection, HEK293A cells were re-plated at 48 h. system. Plasmid construction and establishment of stable cell lines are described in Supplementary Methods. The protocols for both genetic transformation experiments were approved by and performed under the guidelines of the Kyoto University Graduate School of Medicine ethics committee (#130181) and the Shiga University of Medical Science (#29-21 and #31-20). Antibodies used in this work are shown in Supplementary Table S2.

### Immunoprecipitation and LC–MS/MS analysis

For LC–MS/MS analysis, SH-SY5Y cells were maintained in DMEM/F12 containing 15% FBS and 1% non-essential amino acids, transfected with FLAG-FUS WT and P525L using the NEPA21 electroporator and cuvettes (Nepagene) and grown on 10 cm^2^ plates for 48 h. Cells were then lysed in lysis buffer [50 mM Tris–HCl pH 7.4, 150 mM NaCl, 0.5% TritonX-100, 10% glycerol, complete protease inhibitor cocktail], incubated on ice for 20 min, and centrifuged at 12,000 × *g* for 15 min at 4 °C. Lysates were immunoprecipitated using an anti-FLAG M2 affinity gel (Sigma-Aldrich) for 3 h at 4 °C. The affinity beads were subsequently washed with wash buffer [50 mM Tris–HCl pH 7.4, 150 mM NaCl, 0.5% TritonX-100, 5% glycerol] three times, and eluted in elution buffer [25 mM Na-HEPES pH 7.4, 100 mM NaCl, 500 μg/ml FLAG peptide] for 10 min at RT. Purified protein complexes were separated using gel electrophoresis and subjected to silver staining to confirm equal protein loading. Each sample was then processed by SDS-PAGE using a 10% Bis–Tris NuPAGE (Life Technologies) with the MES buffer system. The mobility region was excised into 10 equal-sized segments, and in-gel digestion was performed with sequencing-grade trypsin. They were analyzed by nano LC–MS/MS with a Waters M-Class Nano Acquity HPLC system interfaced with a Thermo Fisher Fusion Lumos. Data were searched using Mascot (Matrix Science) and parsed into Scaffold (Proteome Software). Immunoprecipitation assays with HEK293A lysates were performed using the same method. For the RNase A treatment assay, 10 μg/ml RNase A was added to the cleared lysates before incubation with an anti-FLAG M2 affinity gel.

### Subcellular fractionation and percoll density gradient sedimentation

Mitochondrial and cytosolic fractions were isolated using the Mitochondria Isolation Kit for Cultured Cells (Life Technologies) according to the manufacturer’s protocol. Briefly, cells were homogenized in Reagent A and centrifuged at 1000*g* two times at 4 °C for 5 min. Subsequently, the supernatant was collected and centrifuged at 10,000 × *g* at 4 °C for 15 min to obtain an enriched mitochondrial fraction. The supernatant was collected as the cytosolic fraction. The enriched mitochondrial fraction was washed with Reagent C, followed by centrifugation at 10,000 × *g* for 5 min. Pellets were collected as the mitochondrial fraction.

For the detergent insolubility assay, HEK293A cells were lysed in RIPA buffer [20 mM HEPES–KOH [pH 7.4], 125 mM NaCl, 2 mM EDTA, 1% Nonidet-P40, 1% sodium deoxycholate] containing a protein inhibitor cocktail, incubated on ice for 20 min, separated into RIPA-soluble or -insoluble fractions after centrifugation for 20 min at 15,000 × *g* at 4 °C and subsequently eluted in 2% SDS sampling buffer for 5 min at 95 °C.

For Percoll density gradient sedimentation, HEK293A cells transfected with FLAG-FUS-WT or P525L on 15 cm^2^ plates were homogenized in homogenizing buffer 1 (220 mM mannitol, 70 mM sucrose, 1 mM EGTA, 10 mM HEPES; pH 7.4, complete protease inhibitor cocktail). The homogenates were cleared by centrifugation at 1000 g two times at 4 °C for 5 min and subsequently loaded on 30% Percoll and 0.5% fatty acid-free BSA in homogenizing buffer 1 and centrifuged at 25,000 rpm for 4 °C in a Beckman Type 70.1 Ti rotor. After centrifugation, 15 fractions were collected from the top and used for further analysis.

### SDS-PAGE and BN-PAGE

In SDS-PAGE, the eluates solubilized with 2% SDS were separated on polyacrylamide gels (Wako). Blue-native gel electrophoresis (BN-PAGE) was performed using a NativePAGE Bis–Tris Gel system (Life Technologies). For mitochondrial isolation, cells were homogenized in homogenizing buffer 1 and centrifuged at 1000*g* two times at 4 °C for 5 min. Subsequently, the supernatant was collected and centrifuged at 10,000 × *g* at 4 °C for 15 min, and the pellet was washed in homogenizing buffer 2 (250 mM sucrose, 10 mM HEPES; pH 7.4), followed by centrifugation at 10,000 × *g* for 5 min to obtain the enriched mitochondrial fraction. The mitochondrial fraction was solubilized with 1% digitonin in the sample buffer. For the analysis of complexes I and III, 1% dodecyl maltoside was added. Protein concentration was determined using the Bradford assay. Ten micrograms of protein were run on 4–12% Bis–Tris gels. Proteins were then transferred to a PVDF membrane (Millipore), and immunoblot analysis was performed with the indicated antibodies. Densitometric analysis of protein bands was performed using ImageJ software.

### Immunofluorescence and microscopic analysis

Cultured cells were fixed in 4% paraformaldehyde/PBS (pH 7.2) and permeabilized with 0.1% Triton-X100/PBS containing 5% normal goat serum as a blocking agent. Cells were incubated with a primary antibody (4 °C, overnight) and subsequently with a fluorophore-tagged secondary antibody (Alexa; Life Technologies) for 1 h at 25 °C. The cells were counterstained with 4–6 diamidino-2-phenylindole (DAPI). Fluorescence images were obtained using a confocal laser microscope (Leica TCS SP8).

### Human brain tissue

Formalin-fixed human brain tissue samples from a patient with ALS-FUS (16 years old) and one control case (cerebral infarction, 87 years old) without significant motor neuron pathology and clinical history of motor neuron disease were used in this study. The lumbar cords were obtained from the spinal cord to obtain the corresponding portions from each case. This study was approved by the ethics committee of Kyoto University (approval number: R1038). The patient diagnosis was determined based on clinical information and pathological examinations. The experimental methods for single-labeling immunohistochemistry and electron microscopy are described in “Supplementary Methods S1”.

### Mitochondrial translation assay

Pulse labeling of mitochondrial translation products in transfected HEK293A cells was performed as described in detail elsewhere (Sasarman and Shoubridge, 2012).

### Quantitative real-time PCR

Total RNA samples were purified from HEK293A cells using the RNeasy Plus Mini kit (Qiagen), and 1 μg total RNA was converted to complementary DNA using SuperScript III Reverse Transcriptase (Life Technologies) according to the manufacturer’s protocol. The mRNA expression levels were analyzed using real-time PCR Detection Systems (Bio-Rad) and SYBR quantitative PCR kit (Toyobo). Relative mRNA expression was calculated by the standard curve method normalized to ACTB and relative to the control samples. The primers used are shown in Supplementary Table S3.

### Experiments for mitochondrial dysfunction and cytotoxicity

MitoSOX Red mitochondrial superoxide indicator (Life Technologies) and Hoechst-33342 (Life Technologies) were used for mitochondrial ROS production, TMRM (Life Technologies) for mitochondrial membrane potential, ATP-based CellTiter-Glo luminescent cell viability kit (Promega) for intracellular ATP levels, MultiTox-Fluor Multiplex Cytotoxicity Assay Kit (Promega) for cytotoxicity and viability. Fluorescence and luminescence were recorded by Infinite 200 PRO (TECAN). The details of these experimental methods are described in “Supplementary Methods S1”.

### Statistical analysis

Multiple group means were compared using one-way ANOVA with post hoc Tukey’s multiple comparison tests for pairwise comparisons using GraphPad Prism software (GraphPad, La Jolla, CA, USA). Statistical significance was set at P < 0.05.

### Statement of ethical approval

The human samples were taken for the project “Pathological and biochemical studies of neurodegenerative diseases using human autopsy brain and spinal cord” (No. R1038) by the Kyoto University Ethics Committee. Informed and written consents were obtained from all individuals or their guardians before the autopsy analysis according to the Declaration of Helsinki.

## Supplementary Information

Supplementary Information.

## Data Availability

The datasets generated during and/or analyzed during the current study are available from the corresponding author on reasonable request.
