# Supplementary material for: Conformational change of RNA-helicase DHX30 by ALS/FTD-linked FUS induces mitochondrial dysfunction and cytosolic aggregates
Source: Sci Rep. 2022 Sep 26;12:16030. doi: 10.1038/s41598-022-20405-2 (PMC9512926; doi:10.1038/s41598-022-20405-2)
Supplement: Supplementary file 1 — Supplementary Information. [file 41598_2022_20405_MOESM1_ESM.pdf]

Supplementary information

Conformational change of RNA-helicase DHX30 by ALS/FTD-linked FUS induced mitochondrial dysfunction and cytosolic aggregates

Ryota Hikiami[1-3], Toshifumi Morimura[4], Takashi Ayaki[3], Tomoyuki Tsukiyama[4, 5], Naoko Morimura[6], Makiko Kusui[1], Hideki Wada[1], Sumio Minamiyama[1-3], Akemi Shodai[1], Megumi Asada-Utsugi[1], Shin-ichi Muramatsu[7, 8], Takatoshi Ueki[9], Ryosuke Takahashi[3] and Makoto Urushitani[1, 2 *]

[1] Department of Neurology, Shiga University of Medical Science, Otsu, Japan

[2] Molecular Neuroscience Research Center, Shiga University of Medical Science, Otsu, Japan

[3] Department of Neurology, Kyoto University Graduate School of Medicine, Kyoto, Japan

[4] Research Center for Animal Life Science, Shiga University of Medical Science, Otsu, Japan

[5] Institute for the Advanced Study of Human Biology (WPI-ASHBi), Kyoto University, Kyoto, Japan.

[6] Department of Integrative Physiology, Shiga University of Medical Science, Otsu, Japan.

[7] Division of Neurological Gene Therapy, Center for Open Innovation, Jichi Medical University, Tochigi, Japan.

[8] Center for Gene & Cell Therapy, The Institute of Medical Science, The University of Tokyo, Tokyo, Japan

[9] Department of Integrative Anatomy, Graduate School of Medical Sciences, Nagoya City University, Nagoya, Japan

Supplementary Figure 1-12

Supplementary Table 1-3

Supplementary Methods

Correspondence author Makoto Urushitani, MD, Ph.D.

Department of Neurology, Shiga University of Medical Science, Tsukinowa, Seta, Shiga, Japan Telephone/Fax: +81 (0) 77 548 2160

E-mail: uru@belle.shiga-med.ac.jp

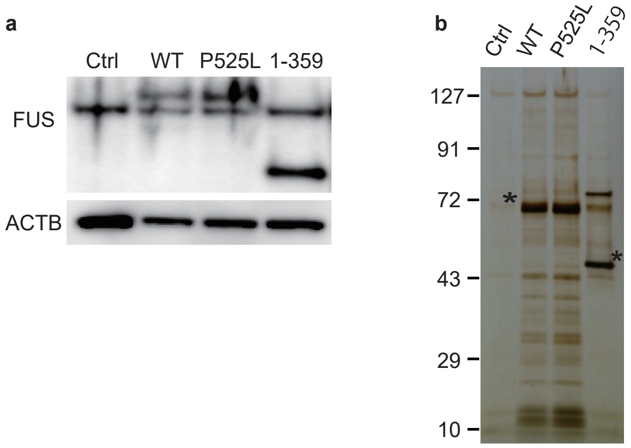

Supplementary Figure S1. related Figure 1. Samples for mass spectrometry analysis of FUS-interacting proteins.

(a) Immunoprecipation was performed using FLAG antibody with SHSY5Y cells transfected with FLAG-tagged FUS WT, P525 L, 1-359, or control vector for mass spectrometry. Western blotting with the indicated antibodies of the inputs. (b) Purified protein complexes were separated using gel electrophoresis and detected by silver staining. The asterisk shows FUS.

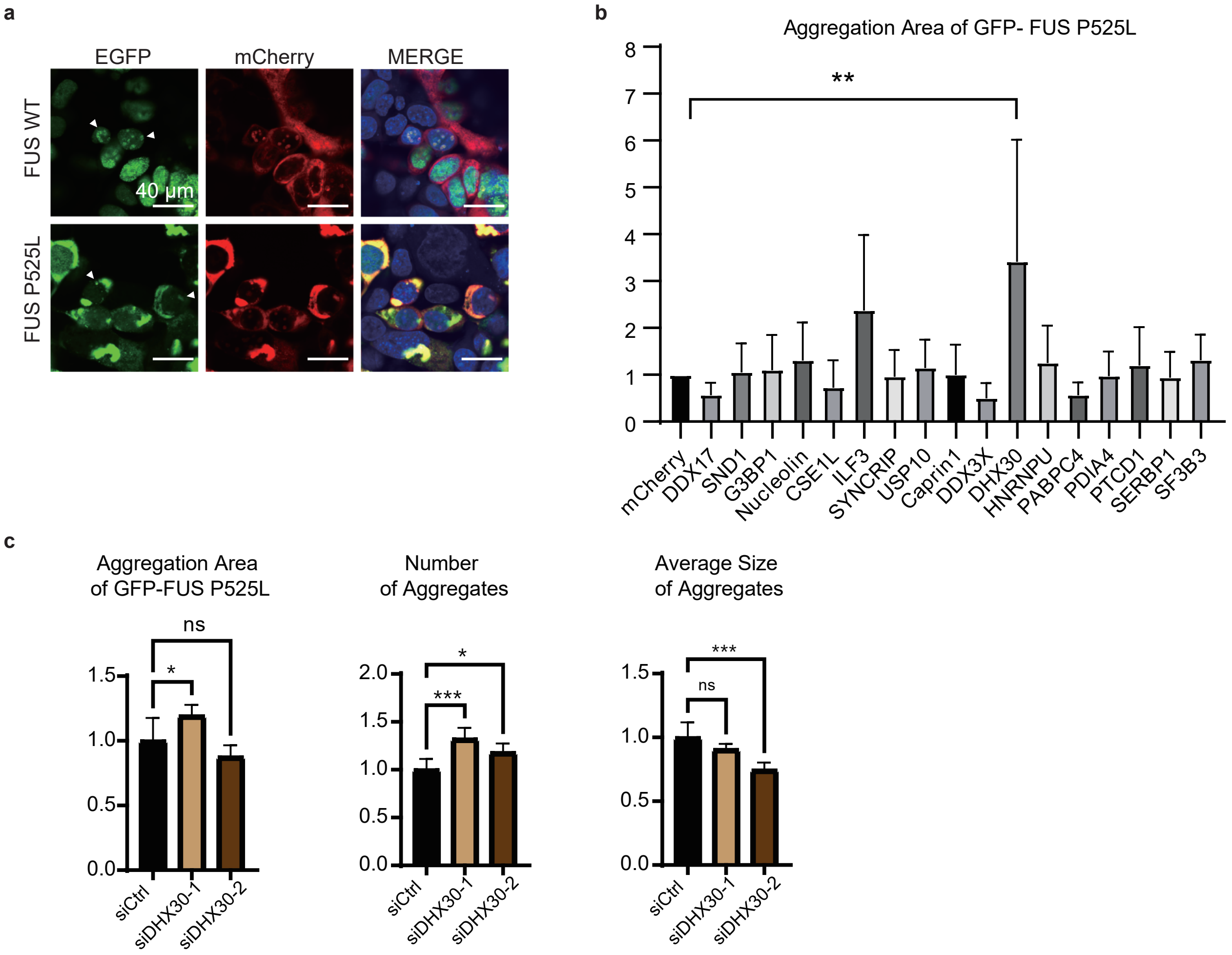

Supplementary Figure S2. related Figure 1. Overexpressed DHX30 strongly colocalized with FUS aggregates and promoted aggregate formation.

(a) Immunofluorescence (IF) assay of HEK293A cells co-transfected with EGFP-FUS WT or P525L and mCherry-DHX30 WT. DAPI was used to counterstain nuclei. IF showed strong co-localization of FUS P525L and DHX30 in the cytoplasm and mild co-localization of FUS WT or P525L and DHX30 in the nucleus, indicated by the white arrows.

(b) HEK293A cells were co-transfected with GFP-FUS P525L and mCherry-tagged interacting proteins with FUS identified in this study, and analyzed at 48h after transfection. n = 4 independent experiments.

(c) HEK293A cells were treated with siRNA-DHX30, transfected with EGFP-FUS P525L at 24h, and analyzed at 48h. n = 6 independent experiments. For quantification of GFP-positive FUS aggregates using ImageJ software, we removed background noise and set fluorescence thresholds to distinguish FUS aggregates from diffusely expressing cytoplasmic non-aggregated species and subsequently counted GFP-positive areas. Each data point was obtained by normalization to mCherry vector or siCtrl. Data were analyzed using a one-way ANOVA with post hoc Dunnett' s multiple comparison tests (*p<0.05, **p<0.01, ***p<0.005, ns = not significant). Error bars represent mean ± SEM.

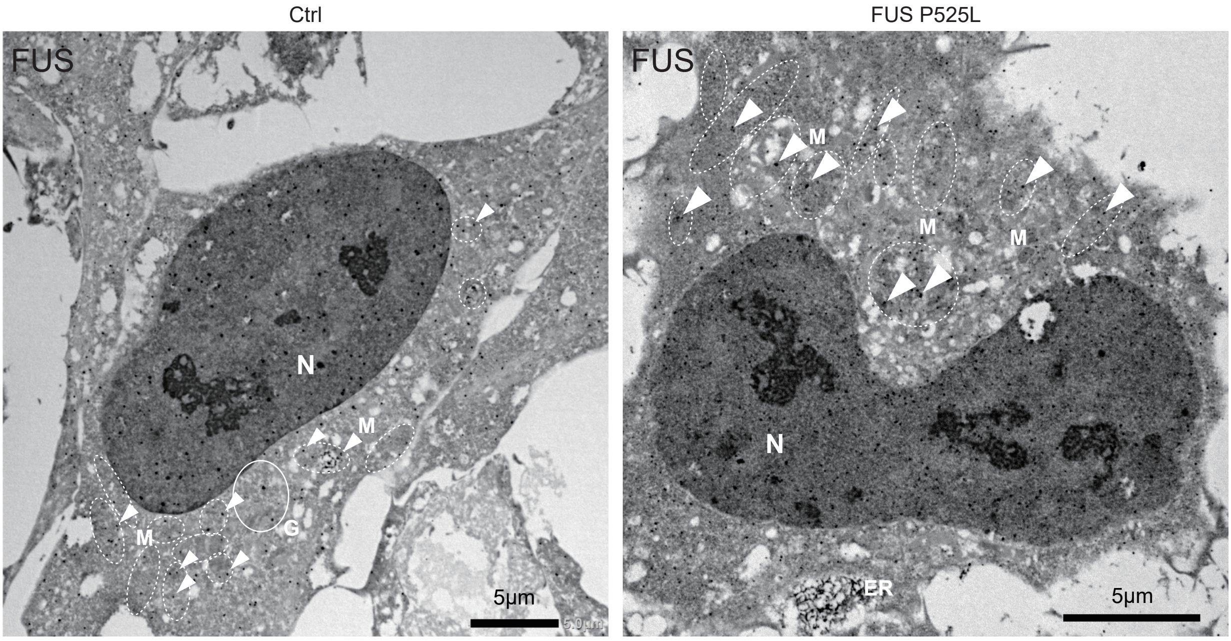

Supplementary Figure S3. related Figure 2. Both endogenous and exogenous FUS were partialy localized to mitochondria in HEK293A cells.

Immunogold electron micrographs for FUS from HEK293A cells transfected with FLAG-FUS P525L or Ctrl shows that both endogenous and P525L FUS are partially localized to mitochondria, more in the mutant. The signal of FUS in mitochondria is indicated by white arrowheads, mitochondria and golgi apparatus are surrounded with white dotted line and white line, respectively.

N: nucleus, M: mitochondria, G: golgi apparatus, ER: endoplasmic reticulum.

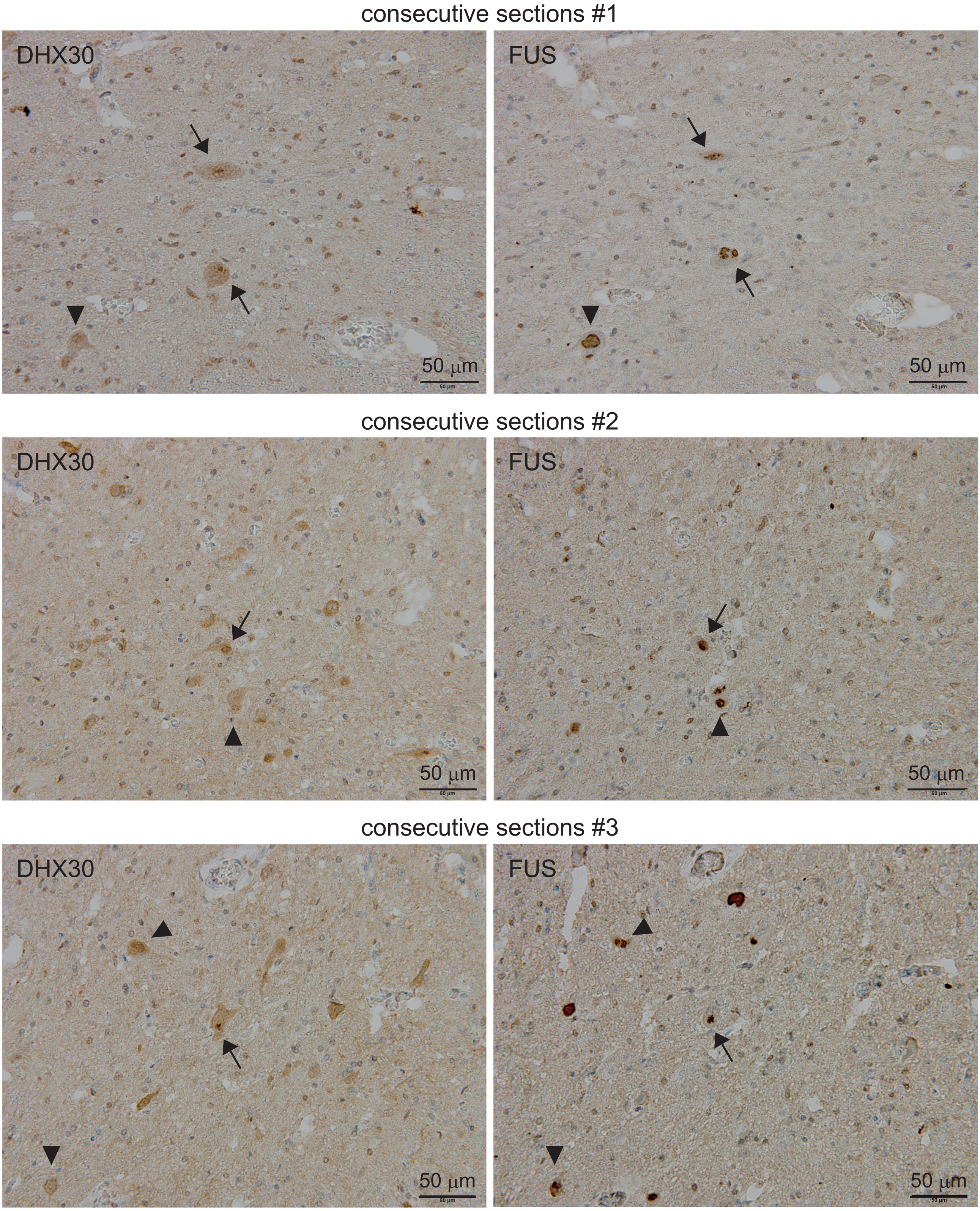

Supplementary Figure S4. related Figure 2. DHX30 colocalized with FUS aggregates in the spinal motor neurons from an ALS-FUS patient.

Representative images of immunohistochemistry using DAB stain for FUS and DHX30 in the spinal motor neurons of the ALS patient with an FUS P525L mutation. Three consecutive sections show clear colocalization of DHX30 and FUS aggregates indicated by arrows, and mild colocalization indicated by arrowheads.

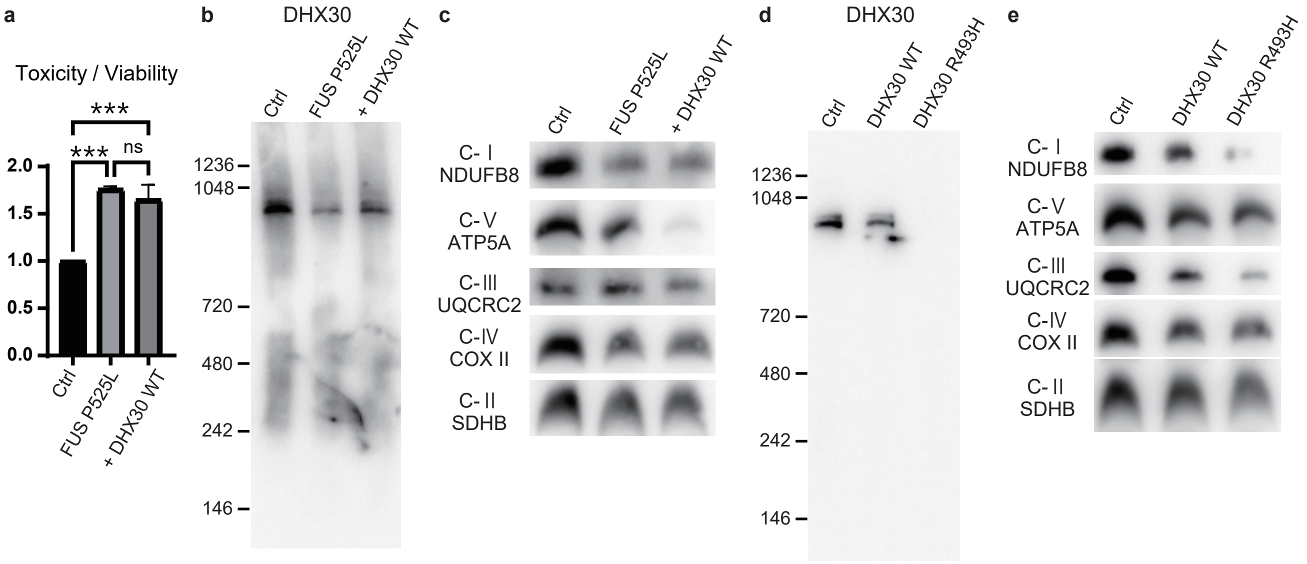

Supplementary Figure S5. Overexpression of DHX30 failed to rescue mutant FUS-induced mitochondrial damage and cytotoxicity.

(a) Quantification of R110/AFC fluorescence ratio of HEK293A cells expressing FLAG-FUS P525L, co-expressing with DHX30 WT, or Ctrl at 96 h. Each data point was obtained by comparison with control. n = 3 independent experiments. Data were analyzed using a one-way ANOVA with post hoc Tukey' s multiple comparison tests (***p<0.005, ns = not significant). Error bars represent mean ± SEM.

(b-c) BN-PAGE analysis with the DHX30 antibody (b) and subunit-specific antibodies against individual OXPHOS complexes (c) of the mitochondrial lysates.

(d-e) BN-PAGE analysis of the mitochondrial lysates with the DHX30 antibody (d) and subunit-specific antibodies against individual OXPHOS complexes (e) of the mitochondrial lysates of HEK293A cells with stable expression of DHX30 WT, R493H, or Ctrl at 96 h.

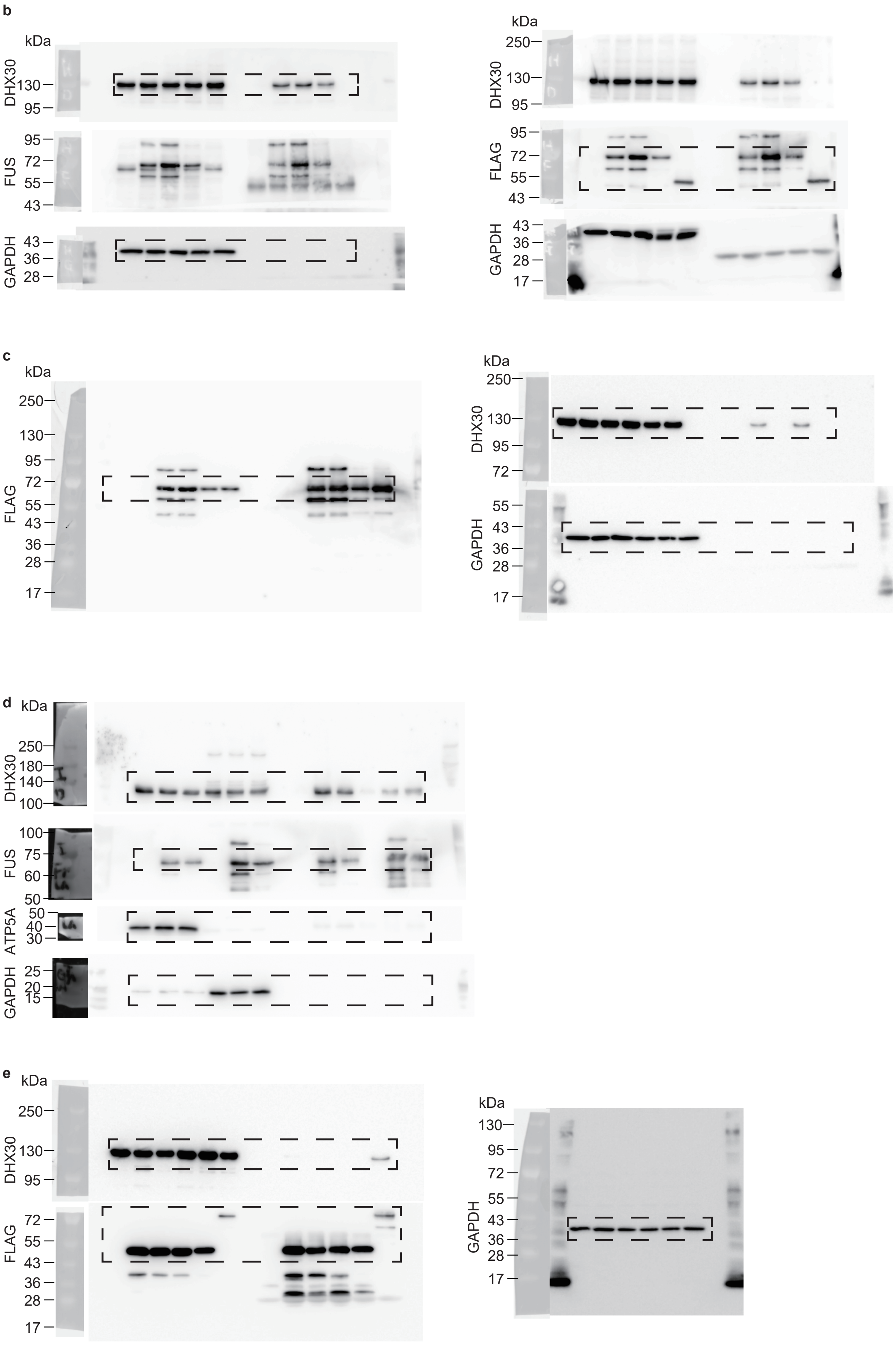

Supplementary Figure S6. Original Western blots of trimmed panels in Figure 1.

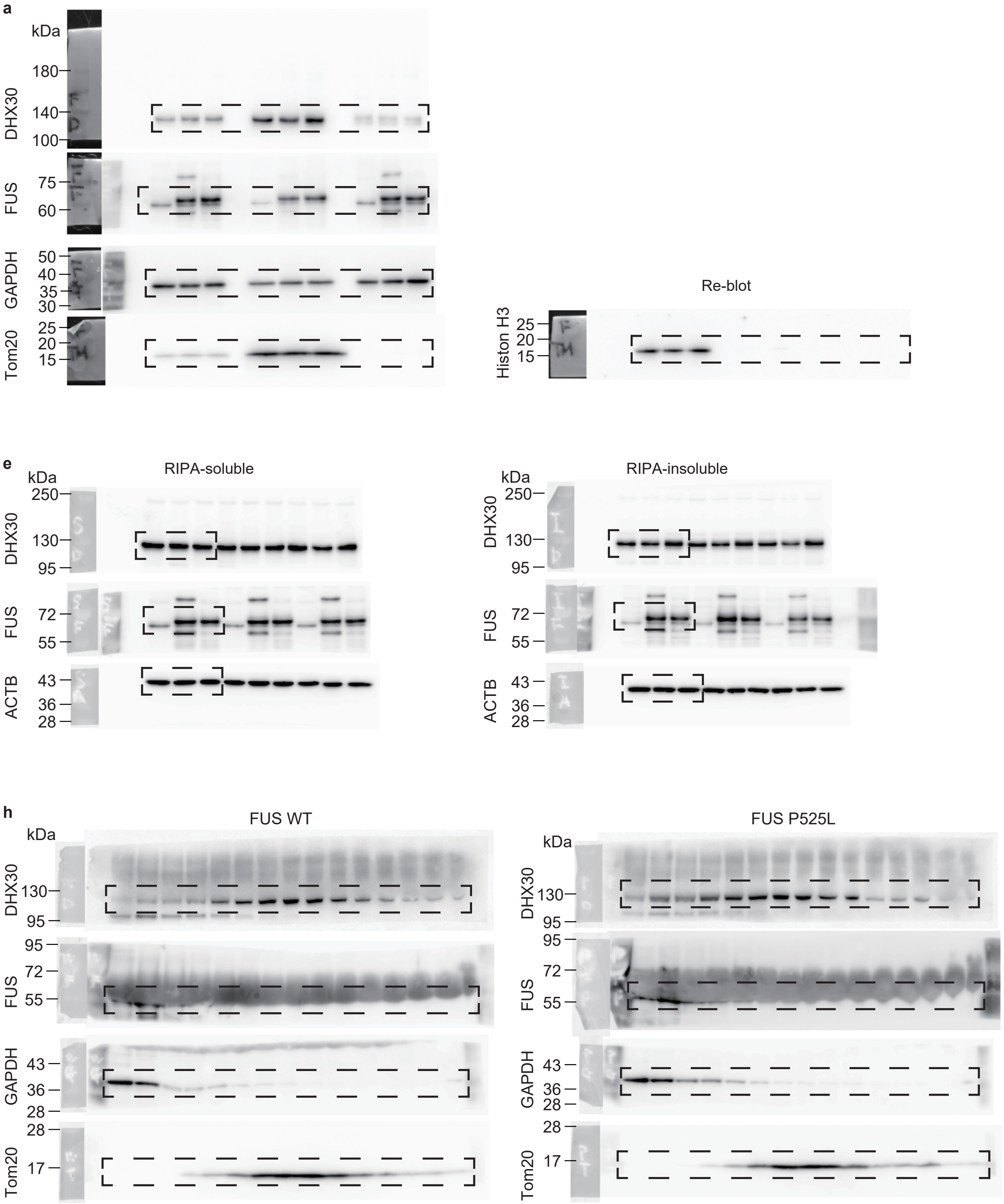

Supplementary Figure S7. Original Western blots of trimmed panels in Figure 3.

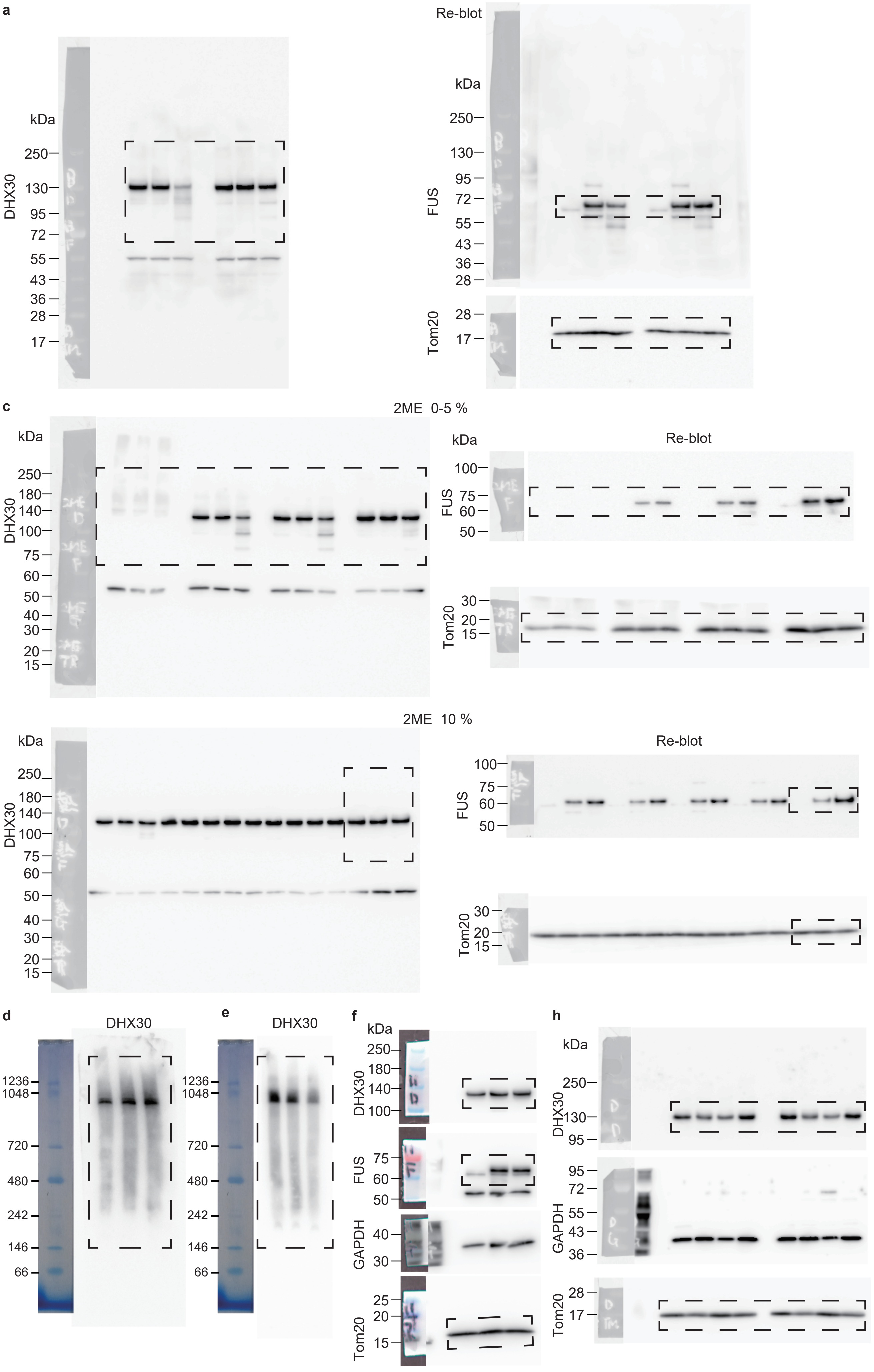

Supplementary Figure S8. Original Western blots of trimmed panels in Figure 4.

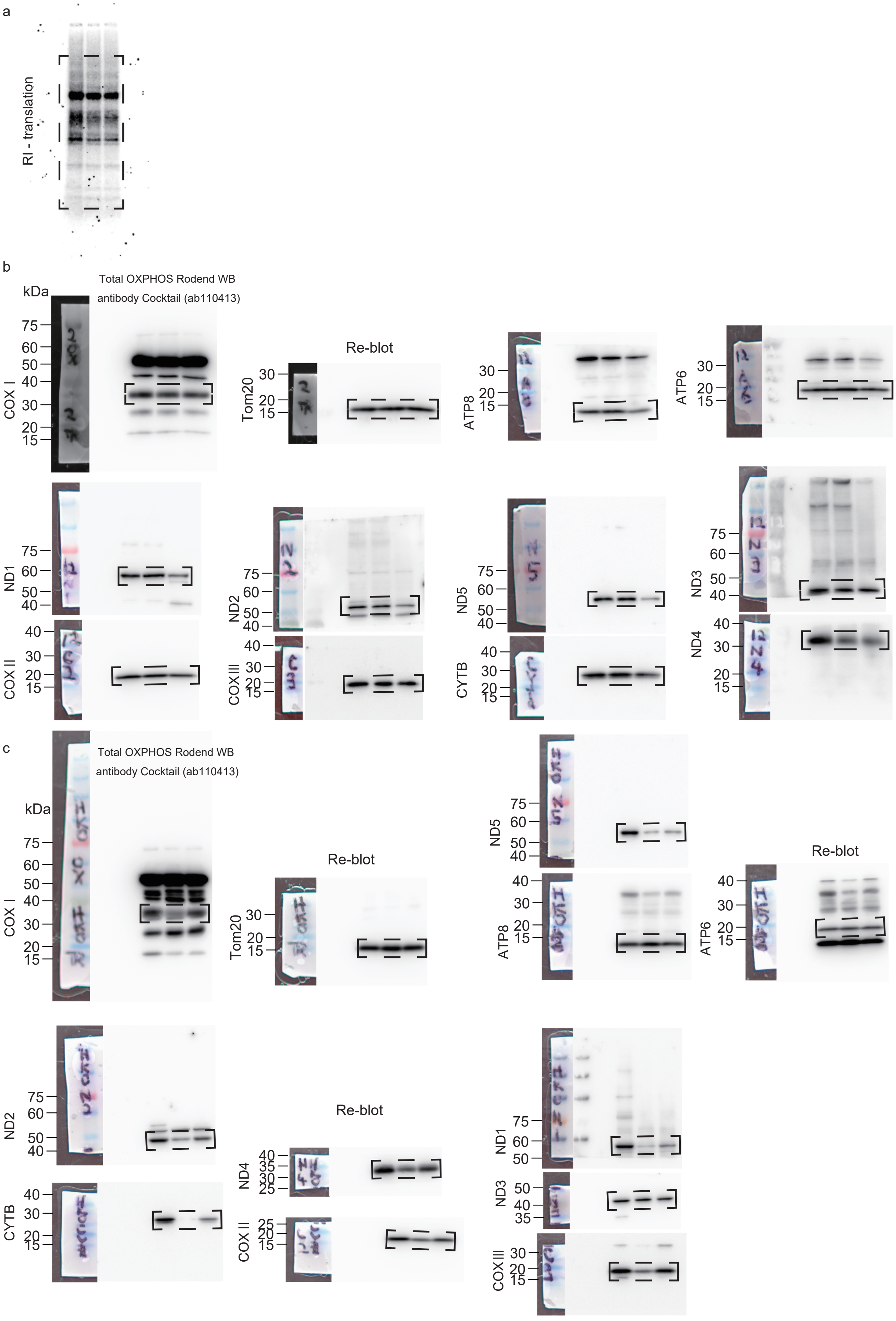

Supplementary Figure S9. Original Western blots of trimmed panels in Figure 5 a-c.

e

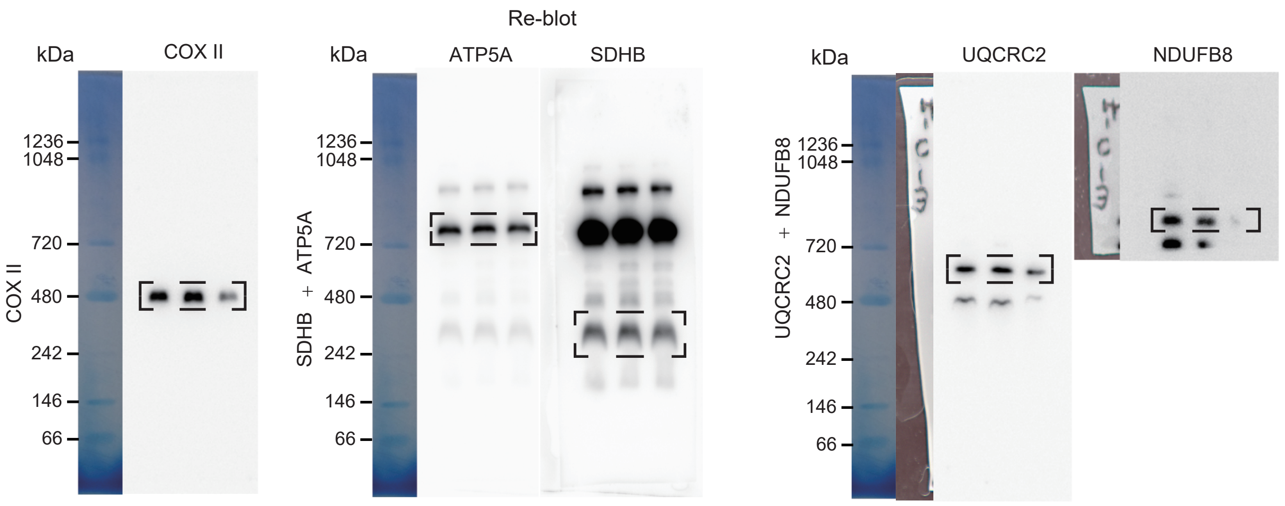

f

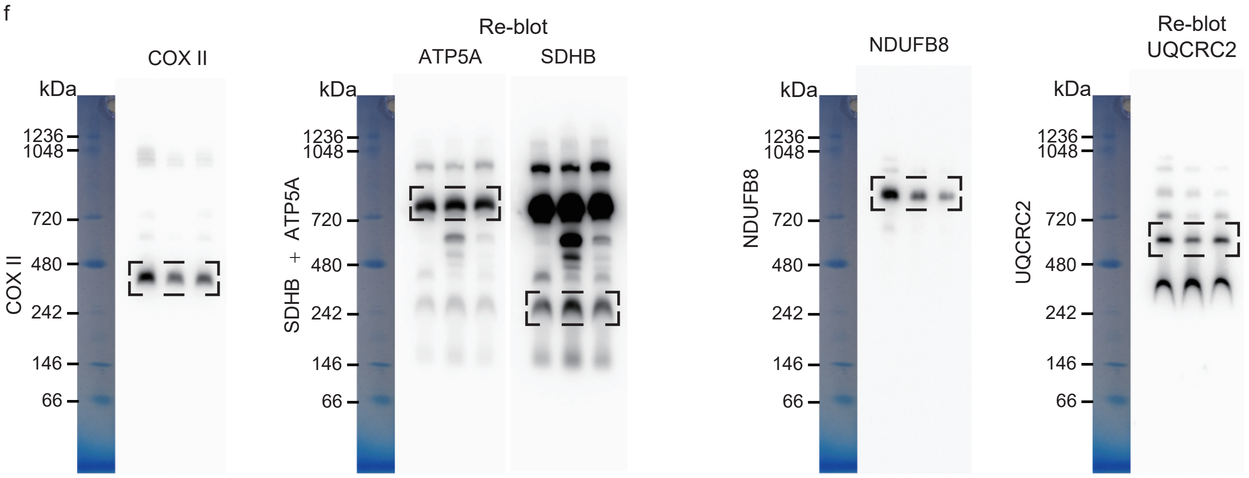

Supplementary Figure S10. Original Western blots of trimmed panels in Figure 5 e-f.

a

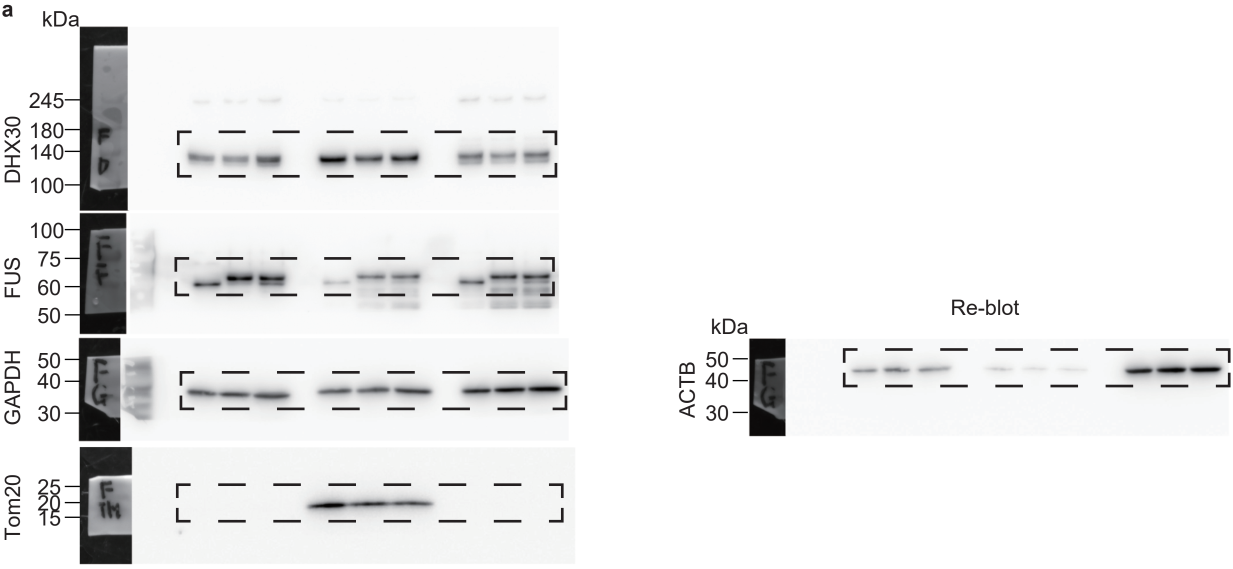

f

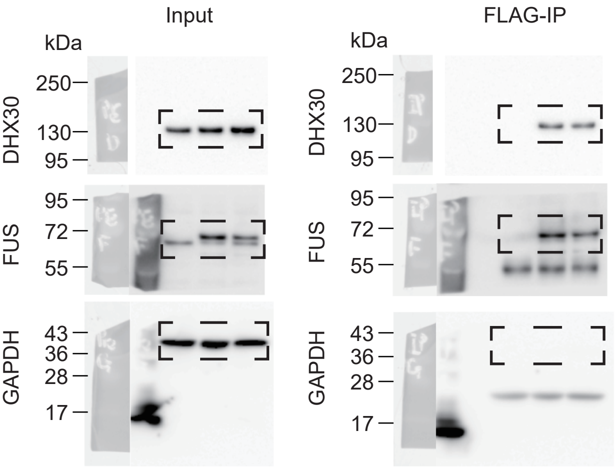

g

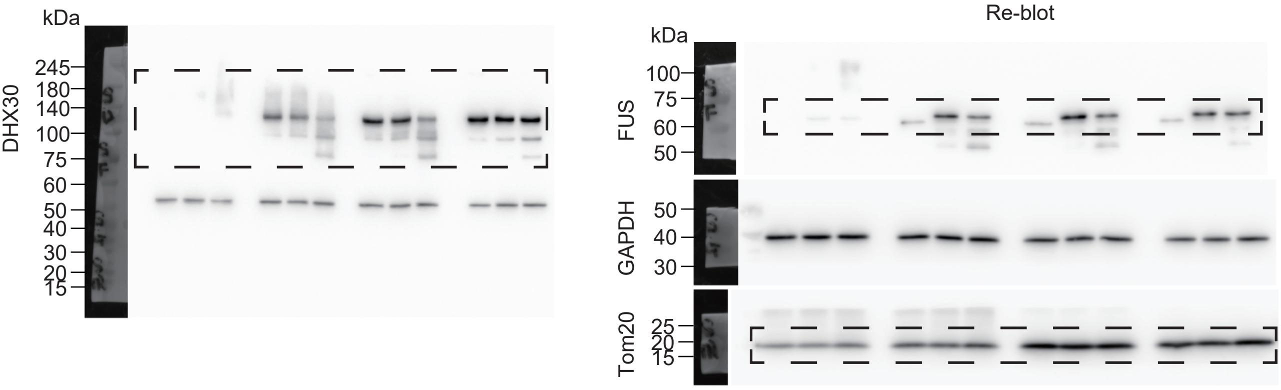

Supplementary Figure S11. Original Western blots of trimmed panels in Figure 6.

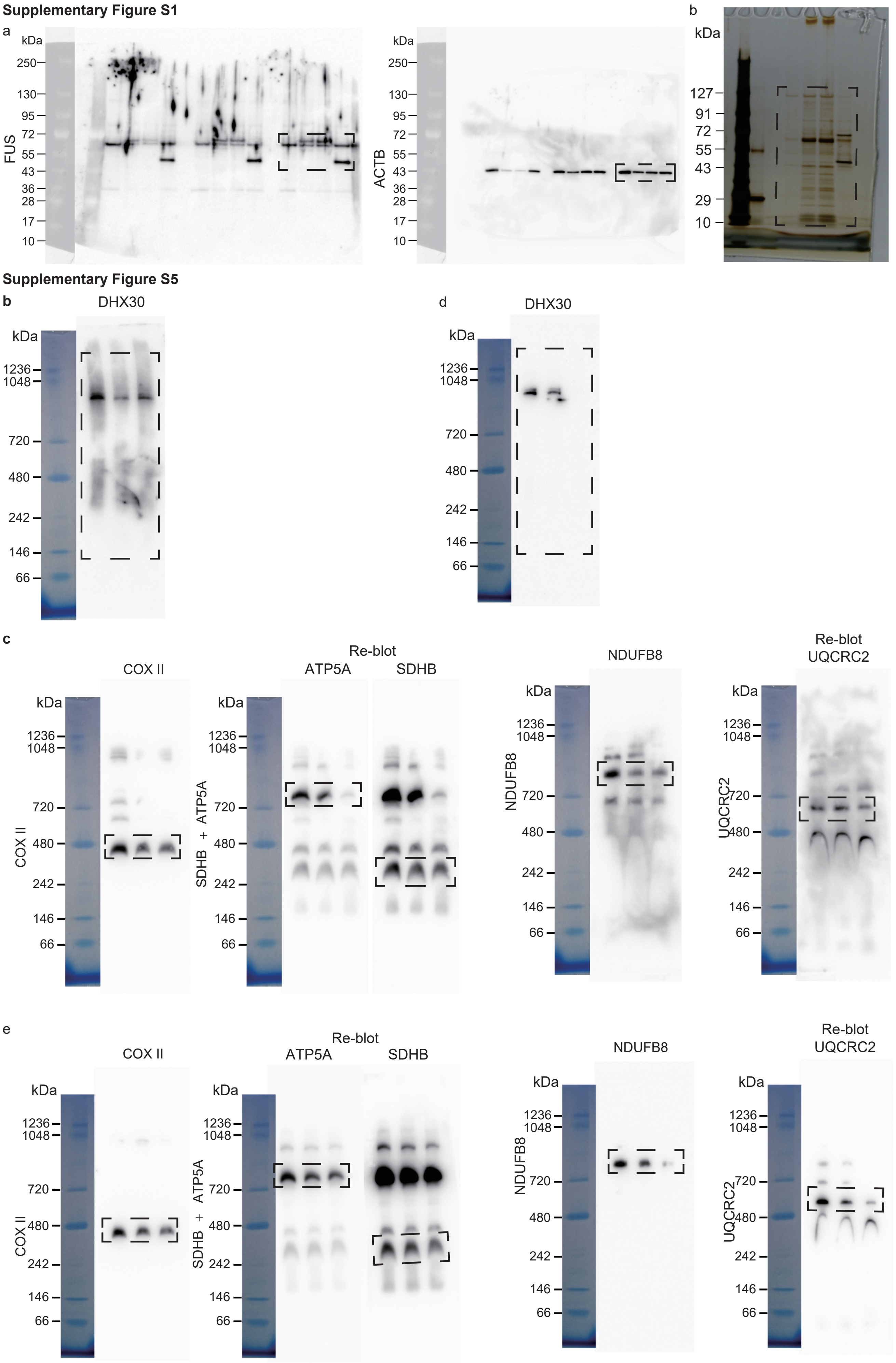

Supplementary Figure S12.

Original Western blots of trimmed panels in Supplementary Fig. S1 and Supplementary Fig. S5.

Supplementary table S1. FUS-interacting proteins.

FUS WT	FUS P525L	FUS 1-359
DNA/RNA binding protein		
FUS	Caprin1	FUS
SYNCRIP	CSDE1	
Caprin1	DDX3X	
RTCB	DDX5	
HNRPA2B1	DHX9	
HNRNPK	DHX15	
HNRNPU	DDX17	
HNRNPUL1	DHX30	
DDX3X	EIF2S3	
DDX5	EIF3B	
DHX9	EIF3L	
DHX30	FUS	
DHX57	G3BP1	
EIF2S3	HNRNPA3	
EIF3B	HNRNPD	
PABPC1	HNRNPH1	
PABPC4	HNRNPH3	
	HNRNPL	
	HNRNPU	
	IGF2BP3	
	ILF3	
	MCM3	
	MCM6	
	NCL	
	NONO	
	PABPC1	
	PABPC4	
	SF3B3	
	SFPQ	
	SND1	
	SNRNP70	

FUS WT	FUS P525L	FUS 1-359
Mitochondrial protein		
ATAD3B	ATAD3B	
ATP5B	LRPPRC	
HADHA	PTCD1	
LRPPRC	SLC25A13	
Chaperone / HSP		
HSPA8	HSPA1A	HSPA1A
HSP9A	HSPA5	HSPA4
HSP90AB1	HSPA8	HSPA8
HSP90B1	HSPD1	
	HSP90AA1	
	HSP90AB1	
	HSP90B1	
	STIP1	
Cytoskeleton		
ACTG	ACTG	JUP
MYH9	EZR	TPM4
SHROOM3	JUP	
TTN	PKP1	
TUBA1B		
TUBB2B		
Modifying enzyme		
PRMT5	GANAB	PRDX1
	PDIA4	PRMT5
	PRMT1	
	PRMT5	
UPS/protein degradation		
	USP10	
	VCP	
Protein transport		
KIF11	AHSG	
	CSE1L	

	SYNCRIP				KIF11	
Ribosomal protein				Others		
RPSA	RPSA			STK38		
RPS3	RPS3			ZC3HAV1		
RPS3A	RPS5					
RPS18	RPS6					
RPS11	RPS8					
RPL4	RPS18					
RPL6	RPS21					
RPL10	RPS25					
RPL22	RPLP0					
RPL28	RPL4					
	RPL6					
	RPL10A					
	RPL11					
	RPL14					
	RPL21					
	RPL27					
	RPL28					

Supplementary Table S2. Antibodies used in study.

	Application	Host	Source	Catalogue#
DHX30	WB	Rabbit	BETHYL	A302-218A
DHX30	IF, IHC	Rabbit	Sigma-Aldrich	outsourcing
FLAG	WB, IF	Mouse	Sigma-Aldrich	F1804
FLAG	IF	Rat	NOVUS	NBP1-06712
Myc	IF	Rabbit	Cell Signaling	#2278
TIA1	IF	Goat	Santa Cruz	sc-1751
MAP2	IF	Mouse	Sigma-Aldrich	MAB3418
GAPDH	WB	Rabbit	Santa Cruz	sc-25778
ATP5A	WB, BN	Mouse	Abcam	ab14748
Tom20	WB, IHC	Rabbit	Proteintech	11802-1-AP
Tom20	IHC	Mouse	Santa Cruz	sc-17764
Histon H3	WB	Rabbit	Cell Signaling	#9717
FUS	WB	Mouse	Santa Cruz	sc-47711
FUS	IHC	Mouse	Proteintech	60160-1-Ig
ACTB	WB	Mouse	Santa Cruz	sc-47778
ND1	WB	Rabbit	Proteintech	19703-1-AP
ND2	WB	Rabbit	Proteintech	19704-1-AP
ND3	WB	Rabbit	Abcam	ab192306
ND4	WB	Mouse	Abnova	H00004538-A01
ND5	WB	Rabbit	Proteintech	55410-1-AP
COX I	WB	Mouse	Abcam	ab14705
COX II	WB, BN	Mouse	Abcam	ab110258
COX III	WB	Rabbit	Proteintech	55082-1-AP
ATP6	WB	Rabbit	Proteintech	55313-1-AP
ATP8	WB	Rabbit	Proteintech	26723-1-AP
CYTB	WB	Rabbit	Proteintech	55090-1-AP
NDUFB8	BN	Mouse	Abcam	ab110242
UQCRC2	BN	Mouse	Abcam	ab14745
SDHB	BN	Mouse	Abcam	ab14714

WB, western blot; BN, blue native PAGE; IF, immunofluorescence; IHC, immunohistochemistry; EM, electron microscopy. outsourcing: This polyclonal antibody was produced against the peptide `CGSFDVRKTADD`, the C-terminal of DHX30, by Sigma-Aldrich.

Supplementary Table S3. Primer sequences for quantitative real-time PCR.

Name	Primer Sequence
12sRNA_F	AACCTCACCACCTCTTGCTCAG
12sRNA_R	ATGGGCTACACCTTGACCTAACG
16sRNA_F	CGTGAAGAGGCGGGCATAAC
16sRNA_R	TGCTCGGAGGTTGGGTTCTG
ND1_F	CGAGCAGTAGCCCAAACAATC
ND1_R	AAGGGTCATGATGGCAGGAG
ND2_F	TCACCCTCCTTAACCTCTACTTCTAC
ND2_R	CGTTGTTAGATATGGGGAGTAGTGT
COX1_F	CCCTCCCTTAGCAGGGAAC
COX1_R	TGAAATTGATGGCCCCTAAG
COX2_F	GATCCCTCCCTTACCATCAAA
COX2_R	GCCGTAGTCGGTGTACTCGT
ATP6_F	CGCCACCCTAGCAATATCAA
ATP6_R	AGGCTTGGATTAAGGCGACA
ATP8_F	ACCACCTACCTCCCTCACCA
ATP8_R	GGGCAATGAATGAAGCGAAC
COX3_F	TGCTTCATCCGCCAACTAAT
COX3_R	AACCACATCTACAAAATGCCAGT
ND3_F	CCCTTACGAGTGCGGCTTC
ND3_R	AGTGGCAGGTTAGTTGTTTGTAGG
ND4_F	CCTCGCTAACCTCGCCTTA
ND4_R	GGAGAACGTGGTTACTAGCACA
ND5_F	AAATCCATTGTCGCATCCA
ND5_R	TTGGTCTAGGCACATGAATATTGT
ND6_F	AAAACAATTTCACAGCACCAAAT
ND6_R	TATGCCTTTTTGGGTTGAGG
CYTB_F	GCGTCCTTGCCCTATTACTATCC
CYTB_R	GCTTACTGGTTGTCCTCCGATTC
ACTB_F	TTCTACAATGAGCTGCGTGTG
ACTB_R	GTACATGGCTGGGGTGTTGA

Supplementary Methods

Plasmid construction and antibodies

Mammalian expression plasmids for FUS tagged with FLAG (pcDNA3-FLAG-FUS) or EGFP (pEGFP-N3-FUS), DHX30 tagged with Myc (pCMV-DHX30-Myc) or mCherry (mCherry-N1-DHX30), and TDP-43 tagged with FLAG (pcDNA3-TDP-43-FLAG) were constructed using a conventional PCR technique, as described previously [1]. The FUS substitution mutant with familial ALS-linked mutations and the TDP-43 substitution mutant with mNLS (R82L/K83Q) or mRRM (C173S/C175S; DCS), and familial ALS-linked mutations (Q331K) were generated by site-specific mutagenesis [2]. Each empty vector was used as the control.

pPB-flox(CAG-Tet3G-IN; TRE3G-cHA-pA), pPB-flox(CAG-Tet3G-IN; TRE3G-DHX30 WT-Myc-pA), and pPB-flox(CAG-Tet3G-IN; TRE3G-DHX30 R493H-Myc-pA) using the TET-ON 3G inducible expression system and the PiggyBac transposon vector system were used for generation of stable cell lines. To generate pPB-floxCAG-Tet3G-IN, loxP sequences and a PCR fragment from pCMV-Tet3G (Clontech) were cloned into pPB-CAG-cHA-IN. To generate pPB-flox(CAG-Tet3G-IN; TRE3G-cHA-pA), pTRE3G-IRES (Clontech) was amplified by PCR and cloned into pPB-hCMV*1-cHA-pA to yield PB-TRE3G-cHA-pA, and then an insert from the vector was introduced into pPB-floxCAG-Tet3G-IN. To generate pPB-flox(CAG-Tet3G-IN; TRE3G-DHX30-Myc-pA), DHX30-Myc was cloned from pCMV-DHX30-Myc and introduced

into pPB-flox(CAG-Tet3G-IN; TRE3G-cHA-pA). To generate pCAG-hyPBase containing PiggyBac transposase, pCMV-hyPBase was cloned into blunt-ended pCAGGS.

To establish stable cell lines, HEK293A cells were co-transfected with pPB-flox(CAG-Tet3G-IN; TRE3G-cHA-pA), pPB-flox(CAG-Tet3G-IN; TRE3G-DHX30 WT-Myc-pA) or pPB-flox(CAG-Tet3G-IN; TRE3G-DHX30 R493H-Myc-pA) and the helper pCAG-hyPBase using FuGENE HD. SH-SY5Y cells were co-transfected with pPB-flox(CAG-Tet3G-IN; TRE3G-cHA-pA), pPB-flox(CAG-Tet3G-IN; TRE3G-FLAG-FUS WT-pA) or pPB-flox(CAG-Tet3G-IN; TRE3G-FLAG-FUS P525L-pA) and the helper pCAG-hyPBase using FuGENE HD. On day 2 post-transfection, the cells were re-plated. On day3, drug selection with G-418 disulfate, 1500 µg/ml in HEK293A cells or 300 µg/ml in SH-SY5Y cells was started. Drug selection was continued for 3 weeks until the surviving cell foci became visible and confirmed by IF. To induce the expression of the stable cell line, 1 µg/mL doxycycline was added to the medium.

Single-Labeling Immunohistochemistry

For single-labeling immunohistochemistry, 6-mm sections were deparaffinized, and antigens were retrieved by autoclaving for 20 min at 120 °C using the Histofine deparaffinizing antigen retrieval buffer, pH 6 (Nichirei), followed by overnight incubation at 4 °C with primary antibodies in PBS containing 3% bovine serum albumin. Single-labeling immunohistochemistry was

performed with antibodies against T.H. and conducted with the peroxidase polymer-based method using the Histofine Simple Stain MAX-PO MULTI (Nichirei) and DAB Substrate Kit (Vector Laboratories).

Electron microscopy for human samples

Samples (1 mm × 2.5 mm) were taken from the superficial portions of tissues embedded in paraffin wax using a sharp-edged carbon steel blade. They were deparaffinized with xylene three times for 10 min at 40 °C, rehydrated with ethanol (100 %, 90%, 70%, 50%) for 10 min each, and rinsed with 0.1 M PBS (pH 7.4) for 10 min. Subsequently, block staining was performed as follows: samples were treated with 10% BSA in PBS for 30 min, incubated with primary antibody against DHX30 (1:500 in PBS with 10% BSA) at 4 °C for 8 h, rinsed with PBS for 10 min three times, and then incubated with nanogold-conjugated secondary antibody (1:100 in PBS with 10% BSA; Nanoprobes) at 4 °C for 8 h.

Furthermore, the samples were rinsed with PBS for 10 min three times, fixed with 1% glutaraldehyde solution in PBS for 1 h, rinsed with distilled water (D.W.) for 5 min three times, and enhanced with silver acetate solution (100 mg silver acetate, 1.4 g sodium citrate・2H2O, 1.5 g citric acid・H2O, 250 mg hydroquinone in 100 ml D.W.). They were incubated in silver acetate solution for 10 min, rinsed with D.W. for 1 min, immersed in 0.05% sodium acetate for 1 min,

rinsed three times with D.W. for 3 min, immersed in 0.05% gold chloride solution in D.W. for 2 min, and rinsed three times with D.W. for 3 min. They were then post-fixed with 0.1% osmium tetroxide in PBS for 30 min and rinsed three times with D.W. for 3 min.

Subsequently, the samples were dehydrated in graded ethanol (50%, 70%, 90%, and 100% three times for 10 min each), substituted with propylene oxide (P.O.) three times for 10 min each, and infiltrated with P.O. and Epon-mixture (1:1, TAAB) for 30 min. They were then polymerized with Epon in polyethylene capsules (6 h at 37 °C, 6 h at 45 °C, and 24 h at 65 °C). Ultrathin sections (70 nm) were cut using Ultracut-E (Reichert-Jung), placed on a collodion membrane-coated 150 mesh copper grid, and stained with 0.2% oolong tea extract (Nisshin EM) solution in PBS for 20 min, 4% uranyl acetate for 20 min, and lead citrate solution (Sigma-Aldrich) for 10 min, and observed under a transmission electron microscope (JEM-2000 FX II, JOEL), operating at an accelerating voltage of 100 kV.

Electron microscopy for cultured cells

The cultures were fixed with 4% paraformaldehyde in phosphate buffer saline (PBS) for 8 h., washed thrice with PBS, and permeabilized with 0.1% Triton-X100 and 1% bovine serum albumin in PBS for 10 mins. After rinsing thrice with PBS, the cultures were incubated in 1% bovine serum albumin in PBS for 30 mins and in primary antibody solution (anti-FUS ab, 1:2000,

1% bovine serum albumin in PBS) for 8 h After rinsing thrice with PBS again, the cultures were incubated in secondary antibody solution (1:100, Nanogold, Nanoprobes Inc, NY, USA, with 1% bovine serum albumin in PBS) for 8 h., rinsed with PBS and enhanced with silver acetate solution for 12 mins67. Following this, the cultures were rinsed thrice with DW, immersed in 0.05% sodium acetate for 1 min, rinsed thrice with DW again, immersed in 0.05% gold chloride solution for 2 mins, and rinsed with DW once more. The cultures were postfixed with 0.1% osmium tetroxide in PBS for 30 mins and rinsed with DW three times. After dehydration of the cultures with graded ethanol and embedding in Epon 812 (TAAB Laboratories, UK), the culture dish was removed, and ultrathin sections (70 nm) were cut parallelly to the cell layers using an ultra-microtome (Reichert-Jung). Sections on 150 square-mesh grids covered with formvar were stained with uranyl acetate, followed by lead citrate, and examined under an electron microscope (JEOL, Japan) at 80 kV.

Mitochondrial ROS production

To determine mitochondrial ROS production, cells were treated with 5 µM MitoSOX Red mitochondrial superoxide indicator (Life Technologies) and 1 µg/ml Hoechst-33342 (Life Technologies) at the end of the experiment for 15 min at 37 °C according to the manufacturer's protocols. Fluorescence was recorded for MitoSOX (excitation (Ex) 510 nm and emission (Em)

580 nm) and Hoechst-33342 (Ex 350 nm and Em 461 nm) using Infinite 200 PRO (TECAN, Männedorf, Switzerland). The MitoSOX fluorescence signal was normalized to the Hoechst reading.

Mitochondria membrane potential measurement

To determine mitochondria membrane potential, cells were treated with 100nM TMRM (Life Technologies) and 1 μg/ml Hoechst-33342 (Life Technologies) at the end of the experiment for 30 min at 37 ºC according to the manufacturers' protocols. Fluorescence was recorded for TMRM (Ex 520 nm and Em 578 nm) and Hoechst-33342 (Ex 350 nm and Em 461 nm) using Infinite 200 PRO (TECAN). The TMRM fluorescence signal was normalized to the Hoechst reading.

ATP measurement

Intracellular ATP levels were determined using an ATP-based CellTiter-Glo luminescent cell viability kit (Promega), which generates a luminescence signal proportional to the amount of ATP present in the cells. Briefly, cells were prepared in opaque-walled 96-well plates. CellTiter-Glo luminescence test solution (100 μl) was added and incubated for 30 min at 25 °C. The luminescent signal was determined using Infinite 200 PRO (TECAN).

Measurement of cytotoxicity and cell viability

Cytotoxicity and viability were determined using a MultiTox-Fluor Multiplex Cytotoxicity Assay Kit (Promega). Briefly, cells were treated with cell-permeant glycyl-phenylalanylamino fluorocoumarin (GF-AFC) and cell-impermeant bisalanyl-alanyl-phenylalanyl-rhodamine 110 (bis-AAF-R110), fluorescent indicators for live cells and dead cells, respectively, at the end of the experiment for 30 min at 37 °C according to the manufacturer's protocols. Fluorescence was recorded for live cells (Ex 400 nm and Em 505 nm) and dead cells (Ex 485 nm and Em 520 nm) using Infinite 200 PRO (TECAN).

1 Urushitani, M., Sato, T., Bamba, H., Hisa, Y. & Tooyama, I. Synergistic effect between proteasome and autophagosome in the clearance of polyubiquitinated TDP-43. *J. Neurosci. Res.* **88**, 784-797, doi:10.1002/jnr.22243 (2010).

2 Tamaki, Y. *et al.* Elimination of TDP-43 inclusions linked to amyotrophic lateral sclerosis by a misfolding-specific intrabody with dual proteolytic signals. *Sci. Rep.* **8**, 6030, doi:10.1038/s41598-018-24463-3 (2018).
